# Metagenomic evidence for reciprocal particle exchange between the mainstem estuary and lateral bay sediments of the lower Columbia River

**DOI:** 10.3389/fmicb.2015.01074

**Published:** 2015-10-01

**Authors:** Maria W. Smith, Richard E. Davis, Nicholas D. Youngblut, Tuomas Kärnä, Lydie Herfort, Rachel J. Whitaker, William W. Metcalf, Bradley M. Tebo, António M. Baptista, Holly M. Simon

**Affiliations:** ^1^Center for Coastal Margin Observation and Prediction and Institute of Environmental Health, Oregon Health & Science University, PortlandOR, USA; ^2^Department of Crop and Soil Sciences, Cornell University, IthacaNY, USA; ^3^Department of Microbiology, University of Illinois at Urbana-Champaign, UrbanaIL, USA

**Keywords:** metagenome analysis, Columbia River estuary, lateral bay sediments, particle exchange, methane cycling

## Abstract

Lateral bays of the lower Columbia River estuary are areas of enhanced water retention that influence net ecosystem metabolism through activities of their diverse microbial communities. Metagenomic characterization of sediment microbiota from three disparate sites in two brackish lateral bays (Baker and Youngs) produced ∼100 Gbp of DNA sequence data analyzed subsequently for predicted SSU rRNA and peptide-coding genes. The metagenomes were dominated by Bacteria. A large component of Eukaryota was present in Youngs Bay samples, i.e., the inner bay sediment was enriched with the invasive New Zealand mudsnail, *Potamopyrgus antipodarum*, known for high ammonia production. The metagenome was also highly enriched with an archaeal ammonia oxidizer closely related to *Nitrosoarchaeum limnia*. Combined analysis of sequences and continuous, high-resolution time series of biogeochemical data from fixed and mobile platforms revealed the importance of large-scale reciprocal particle exchanges between the mainstem estuarine water column and lateral bay sediments. Deposition of marine diatom particles in sediments near Youngs Bay mouth was associated with a dramatic enrichment of Bacteroidetes (58% of total Bacteria) and corresponding genes involved in phytoplankton polysaccharide degradation. The Baker Bay sediment metagenome contained abundant Archaea, including diverse methanogens, as well as functional genes for methylotrophy and taxonomic markers for syntrophic bacteria, suggesting that active methane cycling occurs at this location. Our previous work showed enrichments of similar anaerobic taxa in particulate matter of the mainstem estuarine water column. In total, our results identify the lateral bays as both sources and sinks of biogenic particles significantly impacting microbial community composition and biogeochemical activities in the estuary.

## Introduction

Coastal zones worldwide receive significant nutrient inputs from land through rivers. These inputs are modulated by estuaries (Jickells, 1998; Satinsky et al., 2014) with highly variable physical dynamics that are controlled by tides and by river and ocean forcing. Tides and external forcing also influence the composition of estuarine biota and the nature of nutrient fluxes (Blum and Mills, 2012). Organic matter inputs to estuaries are provided by terrigenous sources (runoff from watersheds transported by rivers), submerged aquatic vegetation of fresh/salt water marshes, and planktonic organisms developing in the river-to-ocean continuum (Crump et al., 2012). These inputs consist of labile dissolved and more recalcitrant particulate organic matter (DOM and POM, respectively; Simon et al., 2002; Jiao et al., 2010).

Estuarine water column particles include mineral, biogenic and anthropogenic components from both aquatic and terrestrial environments. These particles are the foci of biologic activity through colonization by bacteria, protozoa, and metazoa involved in nutrient recycling and organic matter decomposition (Zimmermann-Timm, 2002; Crump et al., 2004, 2012; Grossart et al., 2007; Satinsky et al., 2014). Tidally driven cycles of particle deposition and resuspension provide for continuous chemical exchanges between the aqueous phase, suspended phase, and riverbed (Turner and Millward, 2002). Multiple studies have indicated that particle-associated microorganisms are distinct in characteristics related to growth and activities from those in free-living fractions (DeLong et al., 1993; Crump et al., 1999; Simon et al., 2002, 2014; Smith et al., 2013; Satinsky et al., 2014).

The Columbia River estuary is a particle-rich environment (Haertel et al., 1969) that receives a freshwater supply of over 10 million tons of sediment per year to the estuary (Sherwood et al., 1984). In the lower Columbia River estuary, tidally modulated, hydrodynamic trapping of suspended particulate matter at the salt wedge interface produces estuarine turbidity maxima (ETM). These ETM events extend particle residence time in the water column, promoting development of specific particle-associated microbial assemblages that support up to ∼80% of the secondary production in the estuary (Simenstad et al., 1990; Crump et al., 1998, 1999).

The activities of particle-attached microorganisms in the degradation of autochthonous organic matter is speculated to enhance mineralization of more recalcitrant forms of allochthonous organic matter, resulting in a “priming effect” with potential to greatly enhance organic matter transformations (Guenet et al., 2010; Bianchi, 2011; Simon et al., 2014). In the Columbia River estuary, the priming effect is associated with transient algal organic carbon additions, such as perished and decaying freshwater phytoplankton, the major source of POM from spring to fall (Frey et al., 1984; Sullivan et al., 2001; Herfort et al., 2011b). In late summer to early fall, estuarine blooms of the mixotrophic ciliate, *Mesodinium* sp., likely provide an important autochthonous POM source (Herfort et al., 2011a). In addition, relatively low river discharge allows oceanic water masses to enter the estuary and to reach 30–40 km into the navigation (South) channel (Simenstad et al., 1984). During upwelling periods, the tide often carries coastal phytoplankton blooms, another major source of POM in the lower estuary (Kudela et al., 2005; Herfort et al., 2011a; Roegner et al., 2011b; Breckenridge et al., 2014). The complex interplay of various POM fluxes is significant because our previous results suggested that particle origin is a key determinant of microbial community composition in the Columbia River coastal margin (Smith et al., 2013; Simon et al., 2014).

Previous analyses of mainstem bed of the estuary showed that significant quantities of fine sediment particles originated from the Columbia River lateral bays (Sherwood et al., 1984; Simenstad et al., 1984). The tidal-driven flux of nutrients and organic matter from such intertidal marshes is called “outwelling” (Odum, 2000), and microbial activity in the sediments and overlying water has been shown to profoundly affect estuarine respiratory gas fluxes (Cai et al., 1999). The lateral bays, therefore, function as hotspots of organic-matter degradation (Simenstad et al., 1984) delivering significant amounts of reduced chemical species (e.g., methane, ammonium, and dissolved metals) to the lower estuary (Cai et al., 1999; Turner and Millward, 2002; Bräuer et al., 2011; Gilbert et al., 2013).

Molecular evidence suggests that the lateral bays also contribute to microbial composition in waters of the mainstem estuary. Gene expression and metagenome sequencing studies revealed an enrichment of both anaerobic taxa and genes from anaerobic pathways in oxygenated water samples collected in the mainstem estuary, most notably in ETM particles (Smith et al., 2010, 2013; Simon et al., 2014). Those metabolic pathways, which included dissimilatory nitrate reduction, sulfate reduction, and reduction of Mn and Fe oxides (Klinkhammer and McManus, 2001; Smith et al., 2010, 2013; Bräuer et al., 2011), were not expected to occur in the oxygenated water column, and are thought to indicate the presence of anoxic microzones in particles (Smith et al., 2010, 2013; Simon et al., 2014). The associated taxa were not enriched in the euphotic coastal ocean samples, suggesting an estuarine origin. We hypothesize that particle erosion from the lateral bays of the lower estuary might provide a significant contribution of particulate matter to the estuarine water column and be responsible for “seeding” the ETM with particle-attached anaerobic microbes. An initial step toward testing this hypothesis is characterizing microbial communities associated with the lateral bays.

**FIGURE 1:**
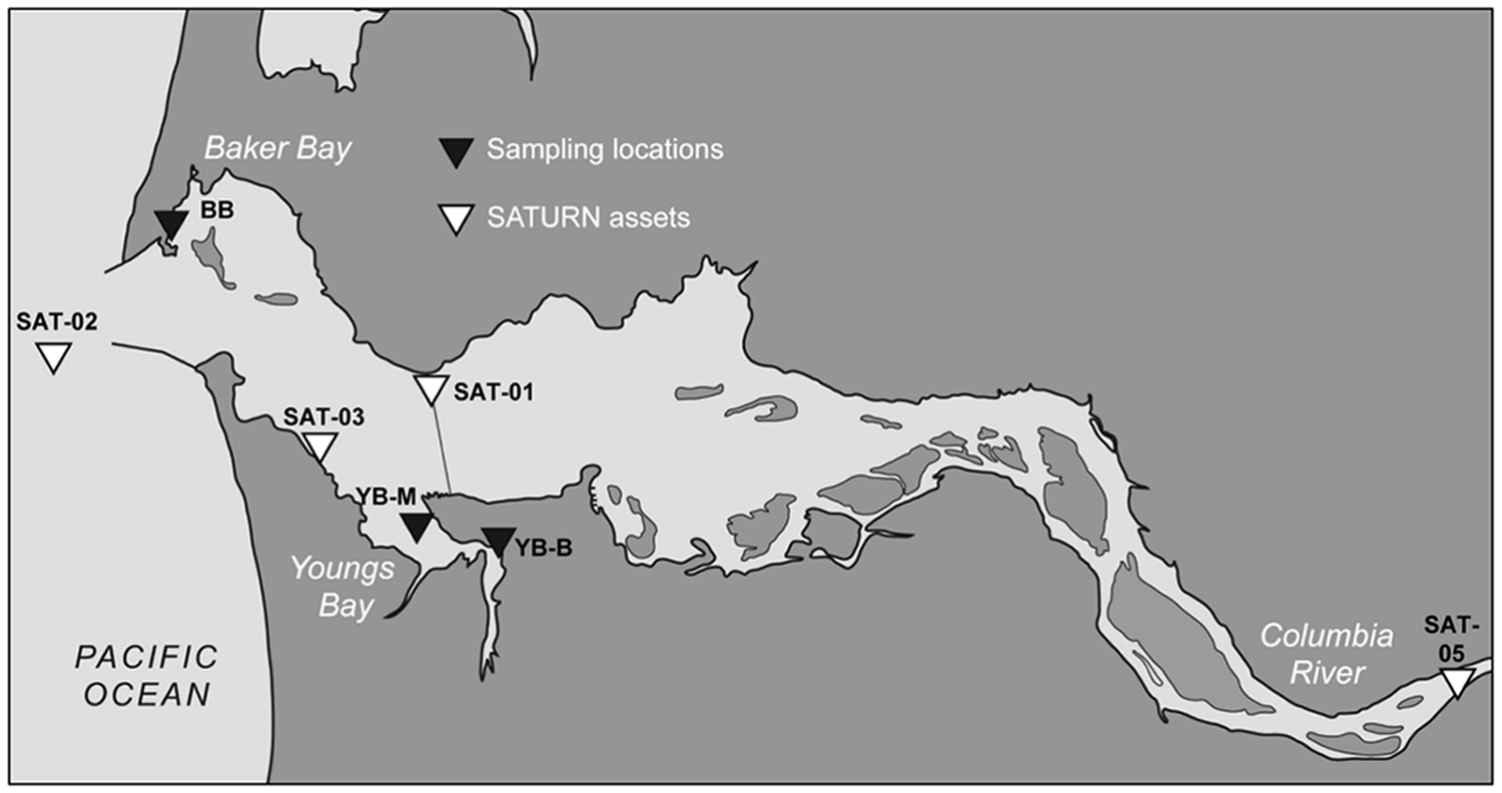
**Contour map of the lower Columbia River estuary.** The shore locations of three sediment sampling sites in Baker Bay and Youngs Bay are shown as black triangles, BB, Baker Bay; YB-M, Youngs Bay mouth, and YB-B, Youngs Bay back. The white triangles show locations of the SATURN endurance stations providing continuous flow-through biogeochemical measurements (including salinity, chl *a* concentrations, etc.).

Our sampling sites were selected to reflect differences in the origin and character of the sediments, as well as in the degree of tidal forcing and salt water intrusion that are observed in these regions. Baker Bay (BB) is a shallow, intertidal inlet located between river miles 3 and 9, with insignificant local tributary inflow (**Figure 1**). It is connected to the North Channel of the main estuary by three conduits, each with dramatic differences in the phase and magnitude of water exchange (Simenstad et al., 1984). The shallow bay interior (including our sampling site) experiences maximum salinities well below ocean values (Simenstad et al., 1984). Youngs Bay is another large, shallow, oligohaline lateral bay, with the mouth located between river miles 11 and 17 at the confluence of the Youngs and Columbia Rivers (**Figure 1**). The Youngs, and Lewis and Clark Rivers provide significant annual local tributary runoff, two-thirds of which occurs in winter, with less than 5% of the total flow occurring in July–September (Simenstad et al., 1984). Salinities at Youngs Bay mouth (at the YB-M sampling location) range from fresh under high river flow, to more than 20 PSU under low-flow summer conditions (Simenstad et al., 1984). Historically, drainage of 1000s of acres of tidal marsh and swamp around Youngs Bay led to enhanced flushing of organic material, silt, and nutrients by the rivers into the bay (Lev et al., 2008). Compared to BB, Youngs Bay is more organic matter rich, supporting a higher abundance of prey for invertebrates, macroinvertebrates, and fish (Bottom et al., 2005).

In this pilot project, we performed high-throughput Illumina sequencing and metagenome analysis to identify the dominant microbial assemblages and pathways involved in carbon transformations in a few selected sediment samples collected just below the surface at three sites in lateral bays of the lower estuary (Baker and Youngs bays; **Figure 1**). Integration of our sequencing data with continuous, *in situ* water monitoring observations from the estuary, plume and coastal ocean together with numerical simulation of water mass transport allowed us to generate testable predictions about the origin and fluxes of particles and their impacts on microbial community composition in the estuary. The study provides important information about the dominant microbial populations and pathways responsible for carbon respiration in the estuary and nutrient exchanges along the river-to-ocean continuum.

## Materials and Methods

### Sediment Sample Collection and Biogeochemical Characterization

Three estuarine sediment samples of ∼50 g each were manually collected on August 22nd, 2011 near shore during low tide in three locations, one in BB and two in Youngs Bay (YB-M and YB-B, Youngs Bay mouth and back, respectively; **Figure 1**). Cores were collected from the wet near-surface sediment (upper 5–10 cm) using sterile 50 mL Corning tubes. Excess water was drained from the tubes before homogenizing and aliquoting sediment in 1 g portions. Total DNA isolation was performed using aliquots stored on ice for 1 day. Frozen (-80°C) aliquots were used to obtain chemical characteristics using Agri-Check (Umatilla, OR), and **Table 1** shows results of bulk chemical analysis for each core.

**Table 1:** Results of bulk chemical analysis of sediment samples.

Sample name	BB	YB-M	YB-B
Lateral bay	Baker Bay	Youngs Bay mouth	Youngs Bay back
Latitude	N46.28551	N46.17469	N46.15968
Longitude	W124.05187	W123.84933	W123.80651
Date	22 August 2011	22 August 2011	22 August 2011
Time (PST)	14:50	13:10	16:20
Class	Sandy loam	Silt loam	Silt loam
Salinity (PSU)^∗^	4–5	2	0
pH	7.5	6.6	6.3
Organic matter (%)	1.8	5.4	8.8
Total P (ppm)	17	6	15
Total Ca (meq)	7.1	10	11.6
Total Mg (meq)	8.1	8.1	10.8
NO3- (ppm)	2	3	2.5
NH4+ (ppm)	15.5	150	170
Total Mn (ppm)	20	243	162
Total Cu (ppm)	2.4	3.6	3.3
Total Fe (ppm)	130	214	254

*^∗^Water salinity data (PSU) are from measurements collected when river discharge levels, phase of the tidal cycle, and temperature data corresponded to those of the sampling date.*

### SATURN Observation Stations and Glider Missions

The biogeochemical data collected by four observation stations of the Center for Coastal Margin Observation and Prediction (CMOP) Science and Technology University Research Network (SATURN) were analyzed for ∼1.5 months prior to sediment sampling. The station locations are shown in **Figure 1**, each station provided near real-time continuous sensor measurements, including salinity, temperature, tidal height, and concentrations of nitrate, dissolved oxygen, and chlorophyll *a* (chl *a*) as previously described (Gilbert et al., 2013). The data are available through the CMOP website (^1^Baptista et al., 2015). The glider missions were performed using the CMOP Slocum 200 m gliders, the instrumentation description, glider tracks, and data are available at http://www.stccmop.org/research/glidermission.

### Numerical Simulation of Water Mass Transport

The likelihood of oceanic waters entering Youngs Bay prior to the sampling time was investigated by running a hydrodynamical circulation model (Zhang and Baptista, 2008) for 1 month starting from August 1, 2011. The model was run at the temporal resolution of 36 s with the model state stored every 15 min (Kärnä et al., 2015). The model was spun up for the first 7 days, and then the simulation analysis was carried out for the period of August 8–23, 2011. Three indicator tracers were added to the simulation to track the fate of riverine, oceanic, and plume water masses. Riverine water mass was defined as water entering the estuary from the tidal river (upstream of Puget Island). Oceanic and plume water masses were defined as water entering from the shelf sea (downstream from the mouth), whose salinity is ≥30 or <30 PSU, respectively. The water age method (Delhez and Deleersnijder, 2002) was used to compute the time that was spent by these water masses in the estuary. In this method each indicator tracer is accompanied by an “age concentration” tracer that accumulates time proportionally to the indicator tracer. The mean age that waters have spent in the estuary can then be computed as the ratio between the age concentration and indicator tracers. Using the simulation outputs, we computed the percentages of the three types of water masses residing in Youngs Bay at any given time, as well as their volumetric fluxes into the bay, and mean age.

### DNA Preparation and Illumina Sequencing

Genomic DNA was extracted from 1 g sediment using the Fast DNA spin kit for soil (Thermo Fisher Scientific, Waltham, MA, USA). DNA concentrations were quantified using the PicoGreen dye (Invitrogen Corp., Carlsbad, CA, USA), on a Nanodrop fluorometer (Thermo Scientific, Wilmington, DE, USA). Approximately 10–30 μg of total DNA was generated from each sample. DNA library preparation and sequencing were performed at the Oregon Health and Science University Massively Parallel Sequencing Shared Resource (OHSU-MPSSR). In total three libraries, each containing DNA from a single sediment sample, were analyzed using Illumina HiSeq 2000 Sequencer (Illumina, San Diego, CA, USA), by 2 × 100 bp paired-end sequencing and the base-calling pipeline (Illumina Pipeline-0.3). Sequencing statistics of the processed data are listed in **Table 2**.

**Table 2:** Metagenome statistics.

General	BB (Baker Bay)	YB-M (YB mouth)	YB-B (YB back)
Number of reads	300,954,858	366,666,206	296,381,839
Individual read length (bp)	101	101	101
Total sequence length (Mbp)	30,400	37,000	30,000
**SSU rRNA**			
Hmmer-selected SSU rRNA reads	55,850	107,241	126,943
Median contig length	219	227	306
Number of selected contigs^∗^	927	1241	1111
% assembled of all SSU rRNA	86.7	87	90.1
Mbp assembled into contigs	4.8	9.4	11.5
Average coverage per contig	13.44X	20.11X	25.96X
**Metagenome assembly**			
Total scaffold length (bp)	537,918,774	322,537,715	278,210,246
# scaffolds	507,501	323,378	303,266
Max scaffold length (bp)	220,933	209,382	373,612
% scaffolds containing ≥2 ORFs	14.9	15.3	14
Median coverage	7.22X	7.72X	10.04X
Median scaffold length (bp)	611	604	587
Effective bacterial genome size	2.71	2.75	2.54
Number of effective genomes	124	81	74
**IMG annotation**			
Gene count	928,712	576,268	524,872
Gene per scaffold	1.83	1.78	1.73
rRNA count	946	1006	824
Phylogeny, % of gene count	69.5	71.4	73.2
Function, % of gene count	58.6	55.9	57.7
COG, % of gene count	58.07	51.09	56.67
Enzyme, % of gene count	20.19	19.18	21.18
Pfam, % of gene count	71.24	68.11	69.85

*^∗^At least 2X coverage, 5 or more reads, contig length >130 bp.*

### Quantitative PCR

Thaumarchaeota *amoA* gene copies were quantified in the sediment DNA using qPCR as previously described (Xu et al., 2012). Standard curves were generated by serial dilutions of linearized plasmids from sediment samples collected on the Washington bank of the Columbia River estuary (105–101 template copies). Melting curves were visually inspected to check for a single peak at expected temperature using MyiQ software (v. 1.0.410, BioRad, USA). LinRegPCR software (v. 11.1.0.0, Heart Failure Research Center, The Netherlands) was used to calculate PCR efficiencies, which were all >1.8. The results were normalized for starting template concentration.

### Pre-assembly Metagenome Analysis of Small Subunit rRNA and Marker Genes

The taxonomic composition of several marker genes was identified and the corresponding taxon abundances quantified in the unassembled metagenomes prior to assembly, to avoid the biasing of abundance metrics by the metagenome assembly pipeline (which is especially pronounced for both rare and very abundant sequences). Specific marker genes, including small subunit rRNA (SILVA rRNA database project (^2^Quast et al., 2013), methyl coenzyme M reductase (*mcrA*), and ammonia monooxygenase (*amoA*), were searched with the HMMER3 software (^3^Finn et al., 2011) using the probabilistic methods of profile hidden Markov models (profile HMMs). The HMMER3-selected reads were then assembled into contigs using Geneious 6.1.5 software (Geneious, Auckland, New Zealand). Each contig was associated with an abundance value expressed as the number of corresponding unassembled 101-bp reads.

For SSU rRNA, from 25 to 127 1000 101-bp reads per metagenome were selected by HMMER3, 87–90% of which were subsequently assembled into contigs (**Table 2**). Contigs were selected for further analysis if they: (i) exceeded 130 bp in length; (ii) were constructed from ≥5 individual reads in both forward and reverse orientations; and (iii) had ≥2X coverage and ≥20 bp overlap between aligned reads. These criteria resulted in ∼1000 SSU rRNA contigs per sample, with a mean coverage of 13.4–26X (**Table 2**). Assembled SSU rRNA contigs were annotated using the CAMERA (Community Cyberinfrastructure for Advanced Microbial Ecology Research and Analysis, camera.calit2.net) Portal with the rRNA Taxonomy Binning workflow, and SILVA databases (Quast et al., 2013). Abundance metrics were preserved for each assembled contig as the number of initial 101 bp reads used for assembly. Thus, the abundance of a taxon identified by SSU rRNA analysis in a sediment metagenome was calculated as a percentage of the unassembled reads corresponding to this taxon over the total number of unassembled SSU rRNA reads.

Two functional marker genes, *mcrA* and *amoA*, were also analyzed using HMMER3 and Geneious 6.1.5 software (Geneious, Auckland, New Zealand). Resulting contigs were annotated using BLAST searches in CAMERA and at the NCBI BLAST portal (Johnson et al., 2008).

### Sequence Data Processing and Metagenome Assembly

Unassembled reads that passed initial QC were filtered using a quality score cutoff of 30 over 95% of the read length, which resulted in removal of ∼45% of the total reads. Normalization of read coverage and read partitioning was performed with khmer v0.6 as outlined in the ‘Partitioning large data sets (50 m+ reads)’ protocol (^4^Pell et al., 2012). Specifically, digital normalization was performed with a k-mer size of 20, a cutoff of 10, and four hash tables, while read partitioning was conducted with a k-mer size of 32, four hash tables, and a minimum hashsize of 128E+9. Read pairs were retained in bins using a custom Perl script available at https://github.com/nyoungb2/metagenome_assembly. Each bin was separately assembled into a scaffold using IDBA_UD v1.1.1 with default parameters (Peng et al., 2012).

Between 300 and 500 1000 scaffolds were assembled, with median scaffold length of ∼600 bp (**Table 2**). Only 14–15% of all scaffolds contained two or more coding sequences (CDS). Because of the assembly process, rare sequences represented by unassembled reads not used in scaffolds were excluded from analysis. For general peptide analysis, there was additional bias in the abundance metrics from loss of information about the number of individual 101-bp reads in the original unassembled metagenome corresponding to each assembled scaffold. This bias was most pronounced for very abundant organisms with 100s of 1000s of hits and deep coverage. However, these problems were outweighed by the improved annotation accuracy achieved with longer sequences. Thus, we used both assembled and unassembled sequence data iteratively to improve overall results from taxonomic profiling of the metagenomes.

### Analysis of Peptide Composition

The assembled metagenomes were analyzed using the Integrated Microbial Genomes (IMG) with Microbiome Samples – Expert Review web server (IMG/M-ER of the DOE Joint Genome Institute^5^). The IMG/M-ER server performed prediction of the most probable RNA coding genes, peptide coding sequences (CDS), and subsequent functional and taxonomic annotations (Markowitz et al., 2008, 2012). Peptide prediction was performed by the IMG/M-ER annotation pipeline via homology [basic local alignment search tool (BLAST)] using the *E*-value cutoff of 10^-5^ as the default. Functional and phylogenetic profiling was done using the IMG collection of reference genomes, and peptide sequence databases of COGs (clusters of orthologous groups), Enzymes, and Pfam (protein families).

### Data Submission and Accession Numbers

The three assembled Illumina datasets are available in the IMG/M-ER metagenome database as a part of the Study with the GOLD ID Gs0047387 (Marine and estuarine microbial communities from Columbia River Coastal Margin). The metagenomes have the following taxon object IDs and project IDs: (i) BB, 3300001371, Gp0055899; (ii) YB-B, 3300001372, Gp0055898; and (iii) YB-M, 3300001373, Gp0056422.

### Metagenome Comparison and Effective Genome Size Estimation

Metagenomes were analyzed by similarity at taxonomic and functional levels. Taxonomic comparison was performed at either (i) the SSU rRNA level using unassembled metagenomes, or (ii) the CDS (predicted peptide) level using assembled metagenomes. For a particular taxonomic category, the relative abundance value was calculated in a metagenome as the sum of rRNA reads or CDS annotated to this taxon divided by the sum total of all rRNA or CDS predicted for the corresponding metagenome (**Table 2**). In some cases (indicated in the text), the abundance values for bacterial families were expressed as percentages of total predicted bacterial CDS. Thus, the significance of a difference between two given samples was calculated based on the two sample *t*-test between percentage values. Functional comparison of metagenomes was done using normalized difference, or *D*-score and *D*-rank calculated as described (Markowitz et al., 2008 available in IMG/M-ER).

Effective bacterial genome size was calculated using a previously defined set of 35 core predicted bacterial marker proteins as described (Raes et al., 2007). The size values were used to calculate the number of effective bacterial genome equivalents for each metagenome (74–124, **Table 2**). Thus, the relative abundance values for functional gene categories of interest were calculated as the total number of peptide hits (CDS) for a category in a given metagenome divided by the number of corresponding bacterial genome equivalents (Smith et al., 2013).

## Results

### Sampling Site Characteristics

Sediment samples were collected from three locations within two lateral bays of the tidally dominated lower Columbia River estuary (**Figure 1**). Two locations, BB and the mouth of Youngs Bay (YB-M), were periodically exposed to oceanic water masses, with near-shore salinities of ≥5 PSU. A third, in the back reaches of Youngs Bay ∼4 km from the mouth (YB-B), represented mudflats in the freshwater interior (**Figure 1**). General physical and biogeochemical characteristics varied greatly between the two bays. BB is characterized by a sandy loam bottom with a high proportion of coarse gravel, and a low percentage of organic matter. In contrast, the Youngs Bay mudflats consist of silt loam bottom sediments, with YB-M and YB-B sediments containing 3–4X more organic matter relative to BB (**Table 1**). At the time of sampling ammonium concentrations were 10 to 12X higher in YB-M and YB-B versus BB, with manganese and iron concentrations at 10-8X and 1.6-2X, respectively (**Table 1**). In general, the Youngs Bay sediment biogeochemistry was consistent with the presence of very active microbially driven organic matter remineralization (Gilbert et al., 2013).

### Biogeochemical Characteristics of the Estuary Prior to the Sediment Sampling

**FIGURE 2:**
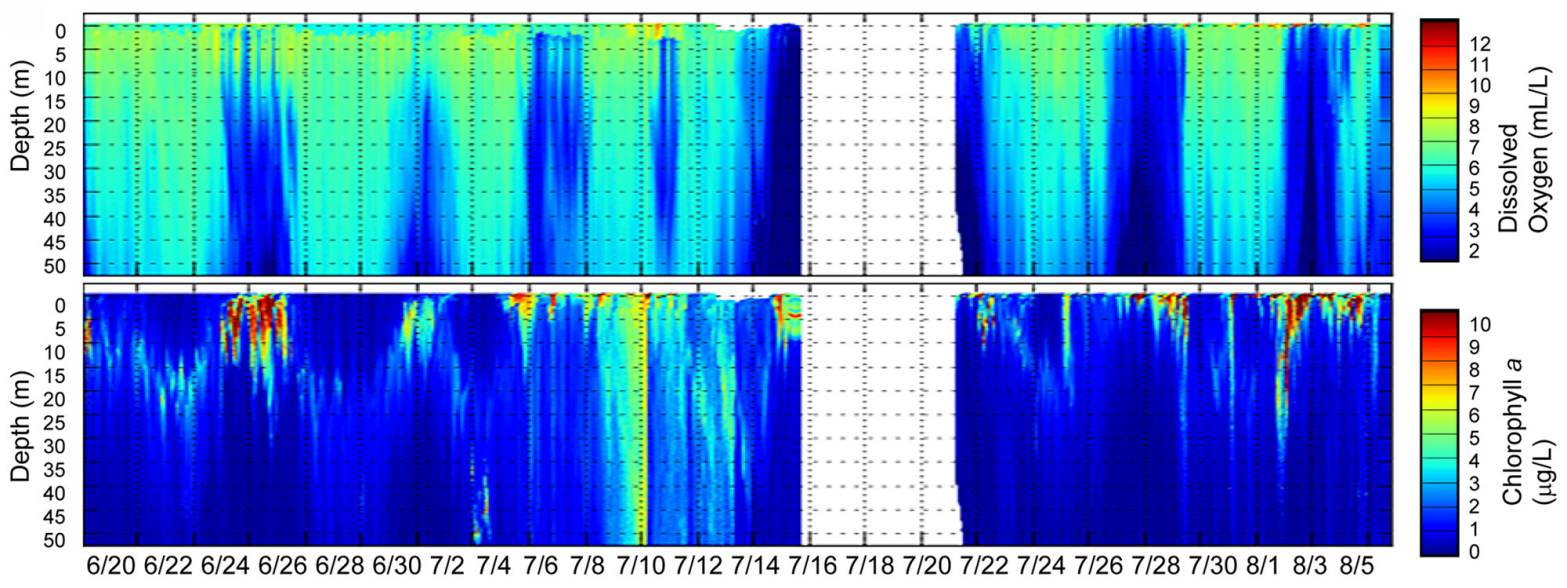
**Glider tracks in the Columbia River coastal margin (two missions between Grays Harbor and Quinault) during the same time period showing (i) the upwelling-related hypoxic zone formation (upper plot with depth values colored by dissolved oxygen values) and (ii) phytoplankton bloom development (lower plot with depth values colored by chl *a* concentrations).** The color scales are shown to the right of each plot. Gaps indicate missing data.

To contextualize our sequence results, we analyzed biogeochemical data collected for estuarine end members (tidal freshwater and coastal ocean) for the period of continuous and pronounced coastal upwelling (data not shown) that started ∼50 days prior to our sampling date in August 2011. This period was associated with persistently high surface chl *a* concentrations in the coastal ocean adjacent to the Columbia River mouth (as observed in near real-time satellite data, Live Access Server of Ocean Watch, The Coast Watch West Coast Regional Node^6^). Sensor data collected during two CMOP glider missions between Grays Harbor and Quinault (June 20–August 6, 2011) indicated widespread, upwelling-related hypoxic conditions at depths between 50 and 175 m, with low-oxygen water reaching the surface at times. In addition, elevated chl *a* concentrations (up to 50 μg/L) were observed, likely indicative of upwelling-related phytoplankton blooms in the euphotic zone (mostly above 20 m depth; **Figure 2**).

**FIGURE 3:**
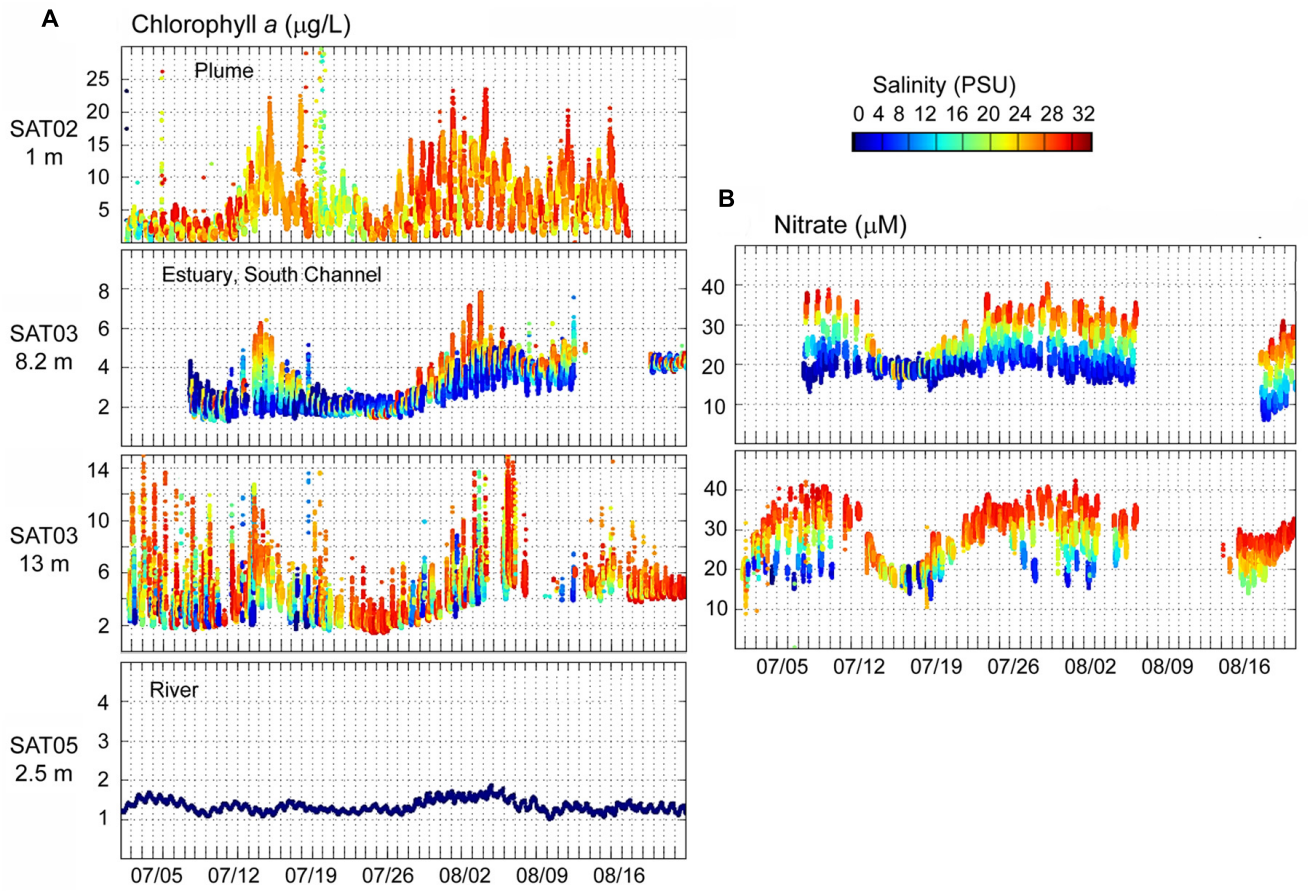
**(A)** Continuous flow-through measurements of chl *a* concentrations (Y-axis, μg/L) at SATURN endurance stations from July 1 to August 20, 2011. Chl *a* values are colored by water salinity (the color scale is shown to the right, in PSU). **(B)** Continuous flow-through measurements of nitrate (Y-axis, μM) colored by salinity. Station names and measurement depths are shown in the upper right parts of the panels. Gaps indicate missing data.

Continuous observation data collected from July 1 to August 22, 2011 by the four SATURN endurance stations (**Figure 1**) were also analyzed. The SATURN-02 (plume) station measured abundant chl *a* in high-salinity water at 1 m depth, in close proximity to the Columbia River mouth (**Figure 3A**, top panel). The estuarine SATURN-03 station in the navigation (South) channel showed a clear association between elevated chl *a* concentrations and high salinity water at all three measured depths (2.4, 8.2, and 13 m; the latter two, having higher chl *a* levels, are shown in **Figure 3A**). Interestingly, the chl *a* peaks in the plume and estuary were observed almost simultaneously (with some exceptions early in July, **Figure 3A**). High salinity water at SATURN-03 contained high nitrate (**Figure 3B**) and low oxygen concentrations typical of upwelled water tidally transported into the estuary (Roegner et al., 2011a,b). In contrast to the high chl *a* observed in the estuary-plume continuum, concentrations at the freshwater Columbia River (SATURN-05) station were very low and did not display the prominent peaks indicative of phytoplankton blooms (**Figure 3A**, bottom panel).

### Water Mass Fluxes between the Mainstem Estuary and Youngs Bay

**FIGURE 4:**
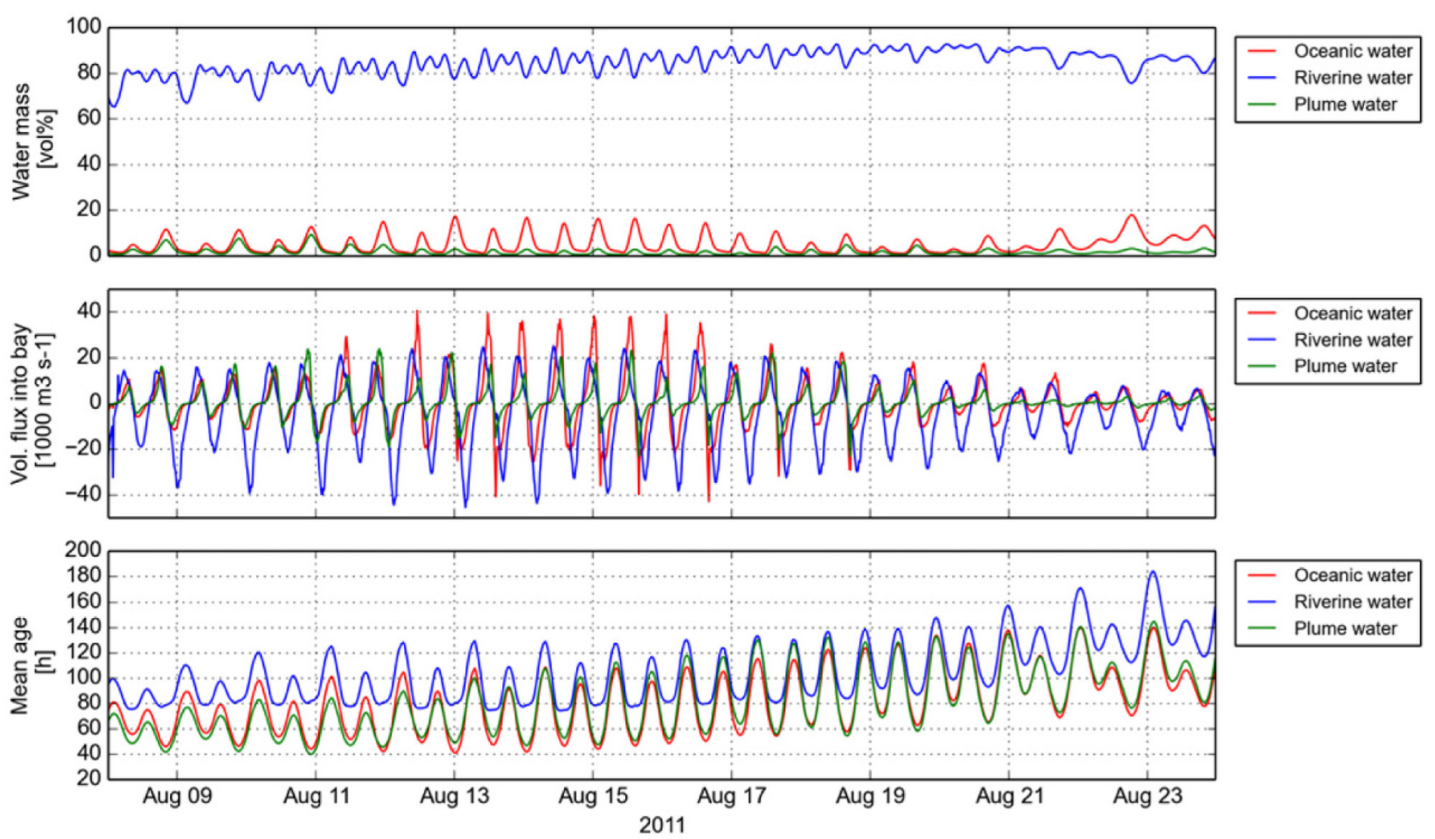
**Numerical simulations of water mass transport in Youngs Bay during a 2-weeks period prior to the sampling time in August 2011.** The plots show the calculated values for (i) fractions of the total volume residing in Youngs Bay **(top)**, (ii) fluxes into the bay **(middle)**, and (iii) mean age of oceanic, riverine, and plume water masses **(bottom)**.

Previous observations indicated that tidally transported oceanic particles could only reach the lateral bay sampling location during late summer/fall periods with correspondingly low river discharges (Chawla et al., 2008). We used numerical simulation of water mass transport during a 2-weeks period prior to the sampling time in August 2011 to evaluate the fluxes, percentages and mean ages of riverine, plume and oceanic water masses residing in Youngs Bay at any given time (**Figure 4**). The results indicated that both oceanic and plume water masses regularly entered the bay, especially during spring tides (August 12–16), when they could occupy up to 18 and 9% of the total volume, respectively. This intrusion was weaker during neap tides (August 19–23), with the daily maximum percentages decreasing approximately twice (**Figure 4**). The mean water age in YB indicated that oceanic/plume waters entering the bay were relatively young during spring tides (less than 40 h after entering the estuary), but the small fraction of water residing in the bay throughout the ebb tides can be considerably older (100 h).

### Metagenome Features

The initial number of unassembled 101-bp reads was similar for all three metagenomes, ranging from 300 to 360 million (**Table 2**). Metagenome assembly reduced the metagenome sizes by approximately two orders of magnitude, in total, from 524 to 929 1000 genes predicted for each metagenome (**Table 2**). Each CDS within a scaffold was considered as a single entry (a predicted peptide) even if it was assembled from multiple reads. We utilized this approach because the majority of scaffolds were short (with the average number of genes 1.73–1.83, **Table 2**), and not particularly abundant, due to high complexity of the sediment microbiota. During the downstream analysis, each predicted CDS of an assembled scaffold was annotated separately and considered as a single independent entry (a peptide). Approximately 70 and 57% of all predicted peptides were associated with taxonomic and/or functional annotations, respectively (**Table 2**).

For each metagenome, the effective genome size was estimated using predicted bacterial peptides (Raes et al., 2007), resulting in values ranging from 2.5 to 2.75 Mbp (**Table 2**). These calculated values were likely underestimates (Konstantinidis et al., 2009), since only annotated bacterial peptides were considered and unclassified CDS were not taken into account. Average GC content of the metagenomes ranged from 41 to 55% with a single, dominant peak, except for the YB-M sample, which had two distinct peaks in %GC distribution: one at 41%, and the second at 61% (data not shown).

### Domain Structure of Sediment Metagenomes

**FIGURE 5:**
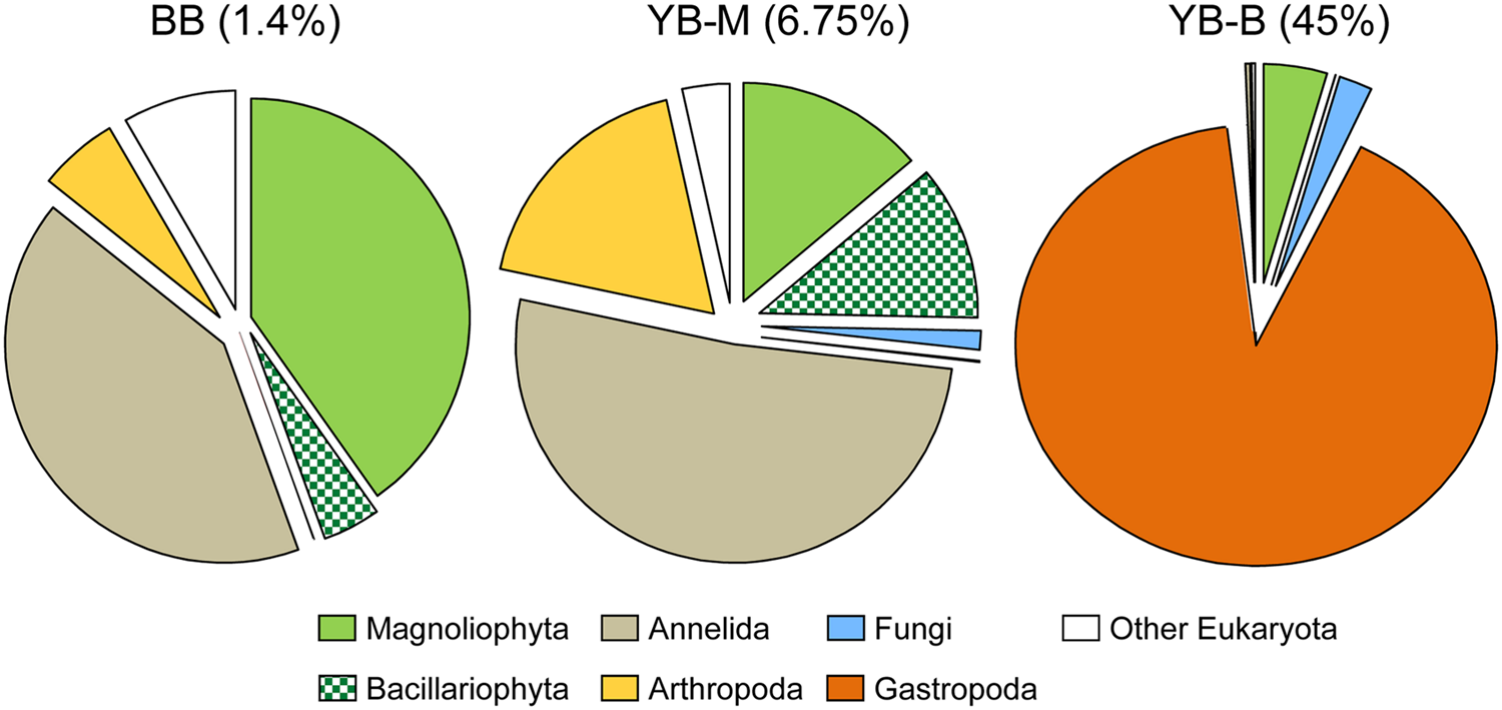
**Taxonomic composition of the domain Eukaryota based on SSU rRNA reads detected by HMMER in the unassembled metagenomes.** The pie charts show taxon abundance, in percentage of total Eukaryotic reads, with color legend given below. Metagenome names indicate sampling locations, BB, Baker Bay; YB-M, Youngs Bay mouth; and YB-B, Youngs Bay back. The numbers in brackets represent percentages of Eukaryotic reads in all of the SSU rRNA reads for each metagenome.

Domain composition of sediment metagenomes was compared using two independent analyses: (i) HMMER3-selected SSU rRNA contigs from unassembled metagenomes, and (ii) predicted peptides (CDS) from assembled metagenomes (**Table 3**). Comparison of SSU rRNA and peptide annotations gave almost identical results for the BB metagenome, with 90% of sequences belonging to bacteria, archaea constituting 8.7% (SSU rRNA) and 8.9% (peptides), and only 1.4% (SSU rRNA) and 1% (peptides) annotated as Eukaryota (**Table 3**). In contrast, the SSU rRNA approach indicated much higher proportions of eukaryotic sequences in Youngs Bay metagenomes in comparison with the predicted peptide approach (7 and 49% of SSU rRNA, versus ∼1.8 and 1.6% of predicted peptides in the YB-M and YB-B, respectively; **Table 3**). The majority of eukaryotic SSU rRNA sequences identified in the YB-B metagenome belonged to Gastropoda mollusks (**Figure 5**). Transient appearance of large numbers of Gastropoda species (up to 200,000 snails/m^2^), namely the invasive New Zealand mudsnail, *Potamopyrgus antipodarum*, was previously observed in Youngs Bay (reviewed in Bersine et al., 2008). The most abundant 18S rRNA contig from the YB-B metagenome (38% of all SSU rRNA reads, 2240X coverage) was 100% identical to the reference *P. antipodarum* SSU rRNA sequence (haplotype 1). The paucity of Eukaryota observed at the peptide level in the Youngs Bay metagenomes (**Table 3**) can be explained by the absence of reference peptide sequence information for large multicellular eukaryotes. In fact, there are currently no sequenced genomes available for Gastropoda, leaving only partial rRNA sequences to serve as annotation references. Apparently, most of *P. antipodarum* peptide sequences from YB-B remained un-annotated, and therefore excluded from downstream analyses. As the result, the total number of peptides predicted for the Eukaryota-rich YB-B metagenome was almost twice smaller than for the Eukaryota-poor BB (**Table 2**).

**Table 3:** Domain composition of the three sediment metagenomes.

SSU rRNA	Bacteria	Archaea	Eukaryota	Chloroplasts
BB	89.63	8.72	1.40	0.25
YB-M	83.08	0.78	6.75	9.40
YB-B	48.93	1.54	49.08	0.45

**Peptide**	**Bacteria**	**Archaea**	**Eukaryota**	**Other**

BB	89.90	8.90	1.09	0.11
YB-M	96.95	1.09	1.78	0.18
YB-B	92.56	5.68	1.64	0.11

*Abundances are expressed as percentages of total number of either SSU rRNA reads detected by HMMER from unassembled sequence reads (upper part), or peptide annotations (CDS with ≥30% identity for ≥70% of the alignment length) generated for assembled metagenomes (lower part). Metagenome names indicate sampling locations, BB, Baker Bay; YB-M, Youngs Bay mouth; and YB-B, Youngs Bay back.*

### Eukaryota

As mentioned above, the YB-B metagenome contained mostly sequences of the Gastropoda *P. antipodarum*, whereas the YB-M metagenome contained predominantly sequences of Annelida (mud worms), Arthropoda (copepods), Magnoliophyta (angiosperms), and Bacillariophyta (diatoms; **Figure 5**). Diatom nuclear-encoded 18S rRNA sequences constituted ∼0.7% of the total SSU rRNA, and most (73%, or 529 hits) were classified as highly identical (98.6%) to the brackish water estuarine taxon *Pleurosira laevis*. In contrast, the highly abundant diatom chloroplast-encoded 16S rRNA sequences (8489 hits, 9.4% of all SSU rRNA sequences, **Table 3**) were mostly (97%) attributed to a different taxon, the pelagic marine diatom *Odontella* (with 99.4% identity over the 1487 bp reference, the *Odontella sinensis* chloroplast 16S rRNA). The peptide data for diatoms did not indicate such high abundance (**Table 3**), most likely because the corresponding reference genomes have not yet been sequenced. Nevertheless, the YB-M metagenome did contain 165 peptide sequences annotated as Bacillaryophyta at the ≥90% level of identity (over at least 70% of the alignment length). Interestingly, nine predicted peptide sequences were annotated as the large subunit of ribulose 1,5-bisphosphate carboxylase (RuBisCo, pfam00016), in addition, 10 corresponded to the photosystem I psaA/psaB protein (pfam00223). Both of these functional gene categories, comprising ∼12% of the identified hits, are chloroplast-encoded. The majority of RuBisCo peptides shared 96–99% identities with the reference sequences from *Odontella, Nitzschia, Cocconeis, Stephanodiscus, Cyclotella*, and *Surirella*, with one sharing 100% identity to the *P. laevis* gene.

### Bacteria

**FIGURE 6:**
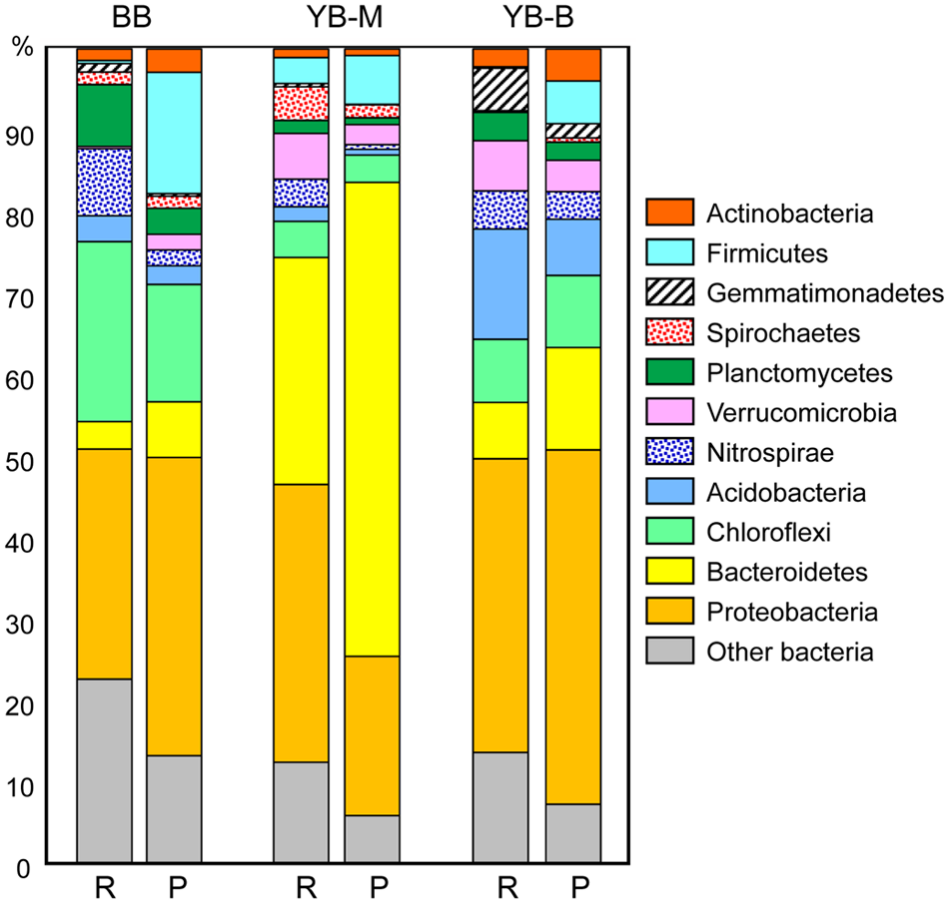
**Phylogenetic composition of the domain Bacteria.** For each metagenome, the left bar (R) represents taxon abundance based on SSU rRNA composition expressed as the percentage of total number of bacterial SSU rRNA reads detected by HMMER in each unassembled metagenome. The right bar (P) represents taxon abundance based on peptide annotations (CDS with ≥30% identity for ≥70% of the alignment length). Each value represents the percentage of all corresponding CDS relative to the total number of bacterial CDS in an assembled metagenome. Metagenome names indicate sampling locations, BB, Baker Bay; YB-M, Youngs Bay mouth; and YB-B, Youngs Bay back.

In all three metagenomes, at least 90% of classified peptides were attributed to Bacteria (**Table 3**). The sediment sequences were more evenly distributed across several abundant bacterial taxonomic groups (**Figure 6**), instead of being dominated by Proteobacteria and Bacteroidetes phyla as observed for metagenomes from Columbia River estuarine, plume and coastal ocean waters (50 to >85%, Smith et al., 2013). As expected, the sediment metagenomes were also relatively more complex, revealing higher diversity of both predicted SSU rRNA and peptides. The most striking differences in bacterial phyla among the lateral bay metagenomes were (i) a high abundance of Chloroflexi (in particular, Anaerolineaceae) in BB (14% of the total); (ii) relatively high numbers of Verrucomicrobia in both YB-M and YB-B metagenomes (3–4%); (iii) a predominance of Bacteroidetes in YB-M (58%); and (iv) a relatively high proportion of Acidobacteria in YB-B (7%; **Figure 6**).

**FIGURE 7:**
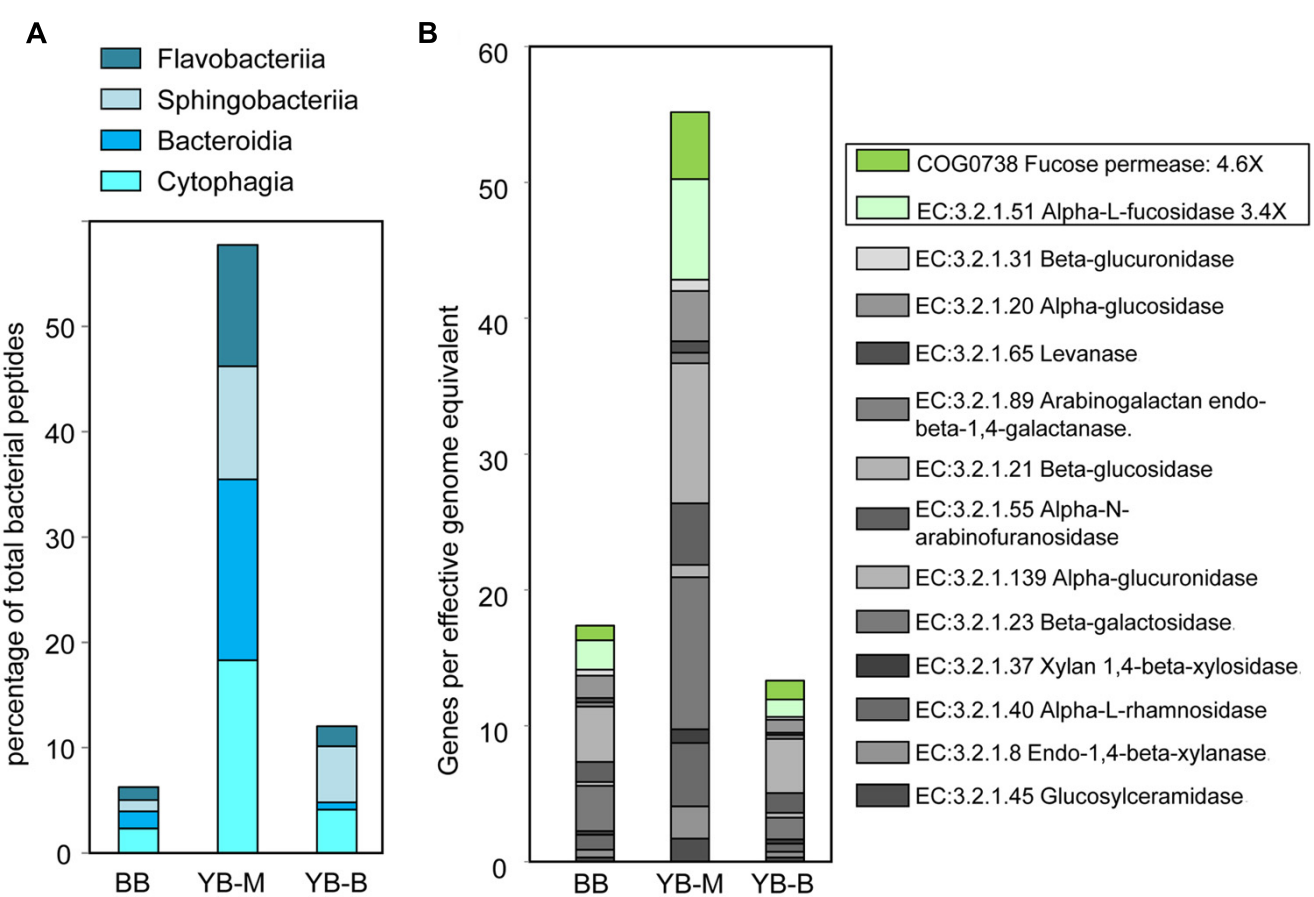
**(A)** Abundance of the four Bacteroidetes classes at the peptide level expressed as the percentage of all corresponding CDS relative to the total number of bacterial CDS in an assembled metagenome. **(B)** Carbohydrate-active enzymes (CAZymes, EC:3.2.1.-Hydrolases. Glycosylases. Glycosidases) showing 14 out of 21 CAZymes with >100 peptide hits that were enriched in YB-M relative to BB and YB-B. Abundance for a functional gene category in an assembled metagenome was calculated as the number of corresponding CDS, normalized by the effective bacterial genome equivalents in a metagenome. Two genes specifically associated with the degradation of diatom cell wall polysaccharides are boxed.

In-depth taxonomic analysis of the phylum Bacteroidetes dominating the YB-M metagenome (58 and 28% of predicted peptides and SSU rRNA, respectively, **Figure 6**) showed higher relative abundance of Flavobacteria (6X), Cytophagia (5X), and Bacteroidia (10X) compared with metagenomes from the other two locations [at both peptide (**Figure 7A**) and SSU rRNA levels; data not shown]. Flavobacteria are known to degrade polysaccharides associated with phytoplankton particles, and therefore contain genes for carbohydrate-active enzymes (CAZymes, EC:3.2.1.-Hydrolases. Glycosylases. Glycosidases; Teeling et al., 2012). Thus, we analyzed the relative abundance of the CAZyme functional gene group, normalized by the number of effective bacterial genomes in each metagenome. A CAZyme gene was selected for analysis if it corresponded to ≥100 predicted peptide hits within a metagenome. We found 21 abundant CAZyme genes (selected from 53 CAZyme genes identified in the sediment metagenomes), 14 of which were enriched ≥2-fold in the YB-M compared to BB and YB-B metagenomes (**Figure 7B**). This enrichment was especially pronounced for fucose permease and α-L-fucosidase (4.6 and 3.4X versus BB, respectively), both of which are associated with utilization of fucose, a major component of diatom exopolysaccharides (Teeling et al., 2012).

**FIGURE 8:**
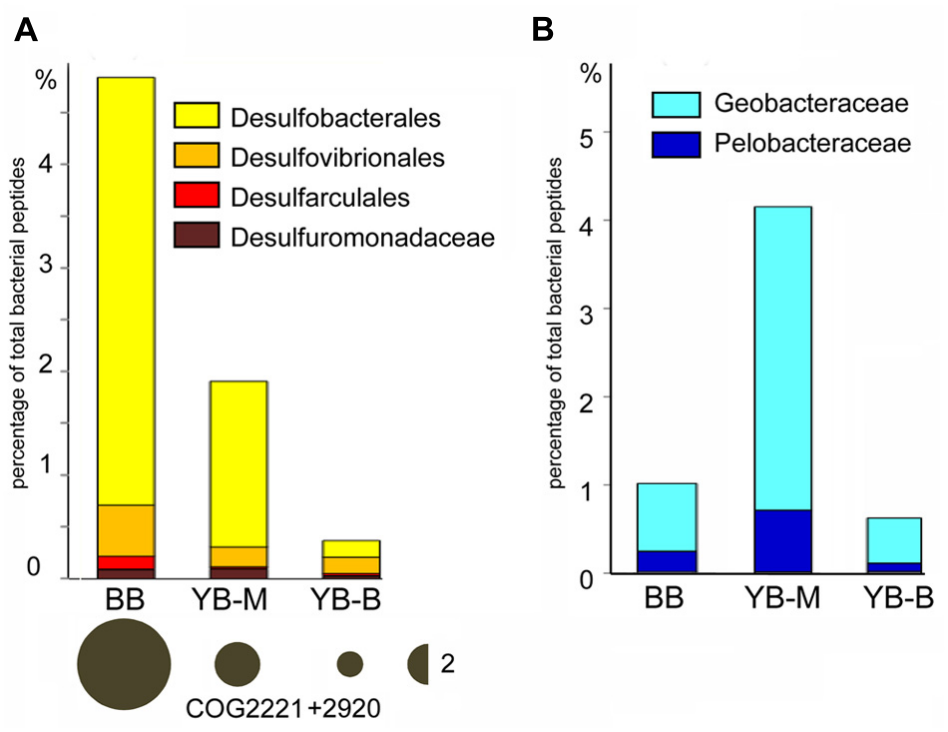
**(A)** Abundance of sulfate reducing bacteria (SRB, bar graph) and the associated marker gene *dsr* (desulfoviridin, bubble plot). Abundance values for SRB were calculated based on peptide annotations at the family/genus level (≥60% identity for ≥70% of the alignment length). Each value represents the percentage of all corresponding CDS relative to the total number of bacterial CDS in an assembled metagenome. Abundance for the *dsr* gene was calculated as the corresponding number of hits, normalized by the effective bacterial genome equivalents in the assembled metagenome. The values are shown with bubble width, the scale is shown as a half-bubble to the right (the number shows the scale value, two genes per the effective bacterial genome equivalent). Metagenome names indicate sampling locations, BB, Baker Bay; YB-M, Youngs Bay mouth, and YB-B, Youngs Bay back. **(B)** Enrichment of Geobacteraceae and Pelobacteraceae (Mn and Fe reducing bacteria). The abundance values were calculated as described above.

The freshwater YB-B sediment contained abundant Betaproteobacteria (15% of predicted bacterial peptides). Further analysis at ≥60% sequence identity (roughly corresponding to family/genus) showed that approximately one half of the corresponding sequences represented various taxa from the order Burkholderiales, whereas another quarter corresponded to the family *Gallionellaceae* (data not shown). In contrast, the two sediments from locations exposed to oceanic water masses, YB-M and BB, were dominated by Deltaproteobacteria, which constituted from 12 (YB-M) to 26% (BB) of all predicted bacterial peptides (at ≥30% sequence identity). At ≥60% identity level, up to 45% of all Deltaproteobacteria sequences belonged to several different orders of dissimilatory sulfate-reducing bacteria (SRB; **Figure 8A**), with highest abundance observed for the families Desulfobacteraceae and Desulfobulbaceae – also prevalent in SRB populations of other brackish to marine estuarine environments (Bahr et al., 2005; O’Sullivan et al., 2013). Consistent with their taxonomic abundance, the marker genes for dissimilatory sulfite reduction, encoding subunits of desulfoviridin (*dsr*), were also enriched in the BB and YB-M compared to YB-B sediments (3.5 and 1.7X, respectively, the bubble plot in **Figure 8A**). In contrast to SRB, other Deltaproteobacteria, namely the families Geobacteraceae and Pelobacteraceae representing the Mn/Fe-reducing anaerobic bacteria, had the highest abundance in YB-M (**Figure 8B**).

### Archaea

**FIGURE 9:**
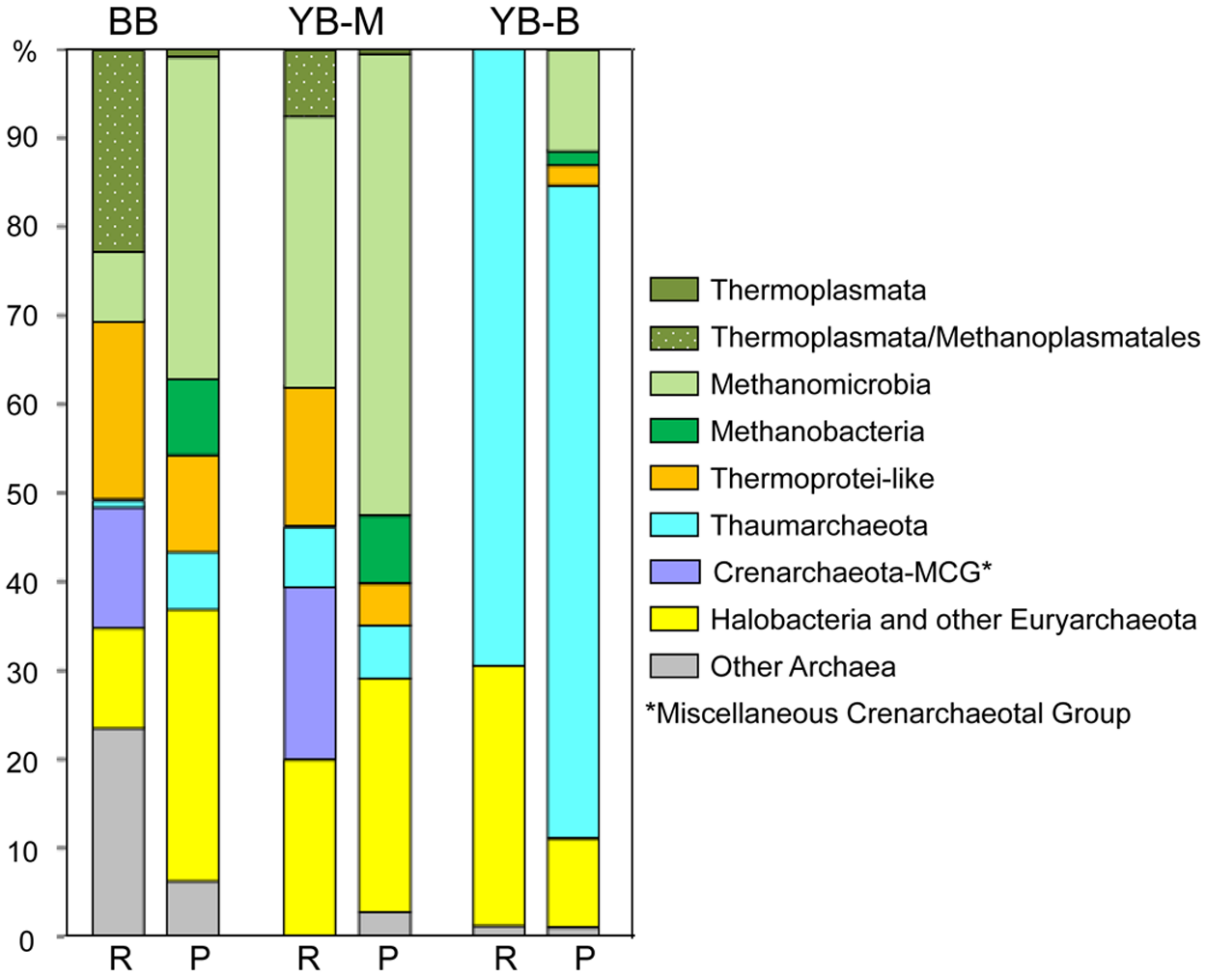
**Phylogenetic composition of the domain Archaea.** For each metagenome, the left bar (R) represents taxon abundance based on SSU rRNA composition expressed as the percentage of total number of archaeal SSU rRNA reads detected by HMMER in each unassembled metagenome. The right bar (P) represents taxon abundance based on peptide annotations (CDS with ≥30% identity for ≥70% of the alignment length). Each value represents the percentage of all corresponding CDS relative to the total number of archaeal CDS in an assembled metagenome. Metagenome names indicate sampling locations, BB, Baker Bay; YB-M, Youngs Bay mouth; and YB-B, Youngs Bay back.

**FIGURE 10:**
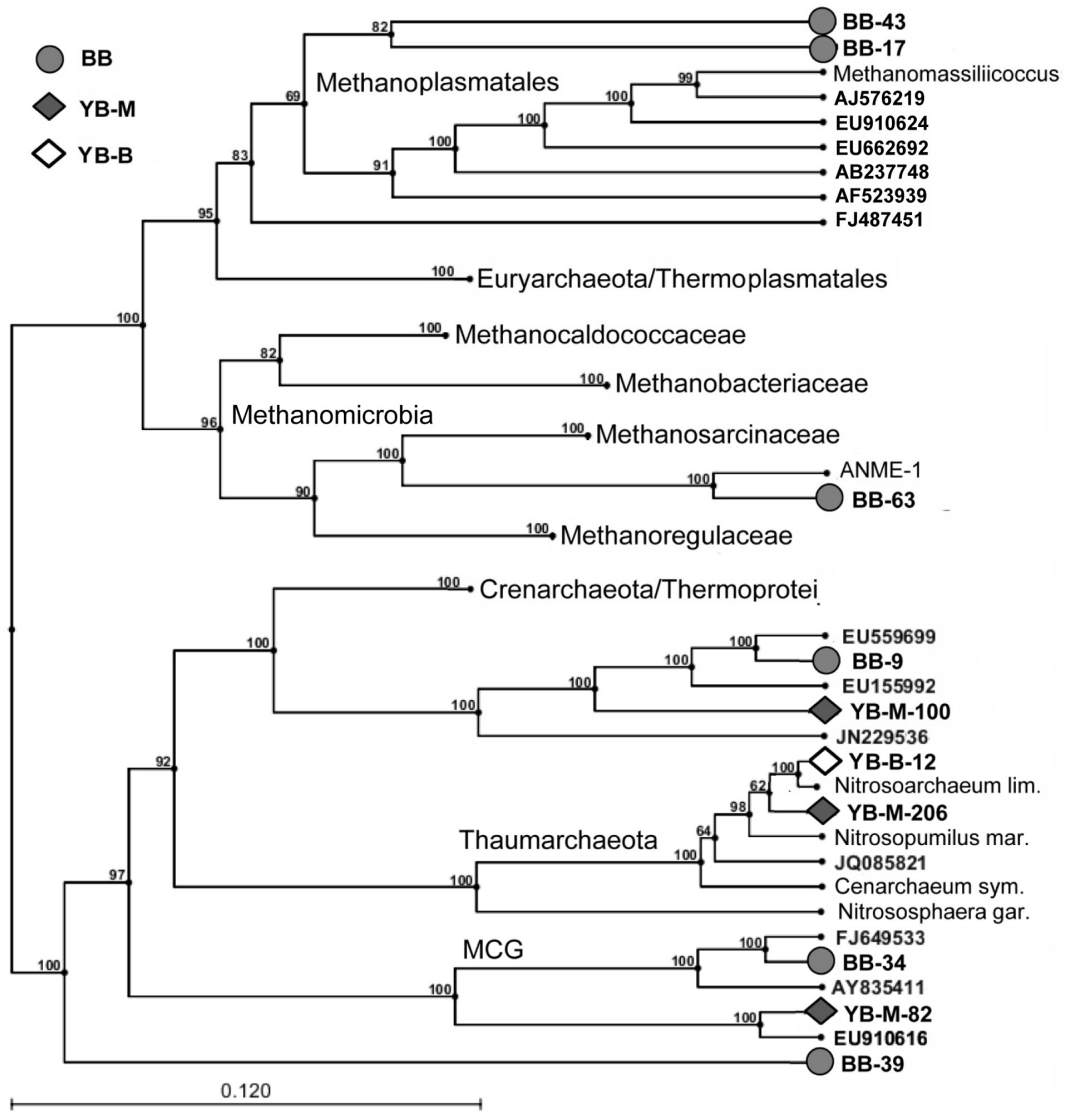
**Inferred phylogenetic tree showing archaeal 16S rRNA contigs from the lateral bay sediment metagenomes (UPGMA, bootstrap analysis with 100 replicates).** Only contigs with at least 4X coverage that were >1 kb in length are shown. Gray circles (BB), gray diamonds (YB-M), and open diamonds (YB-B) indicate contigs from Baker Bay, Youngs Bay mouth, and Youngs Bay back, respectively. Phylogenetic clades were anchored using both rRNA sequences from fully sequenced archaeal genomes (shown with organism names) and selected environmental rRNA clones (most closely related to our sequences and shown with GenBank accession numbers). Numbers represent bootstrap values corresponding to each branch.

The highest abundance of Archaea, constituting ∼9% of all SSU rRNA and predicted peptides, was observed in the BB metagenome (**Table 3**). In YB-M and YB-B, respectively, 0.8 and 1.5% of the archaeal sequences were identified from SSU rRNA analysis, and 1–5.7% from predicted peptides (**Table 3**). Differences in results from these approaches likely reflect a number of issues including: (i) variation in copy numbers of rRNA genes, (ii) relative scarcity of annotated peptide references available for Archaea, and (iii) annotation of archaeal genes as bacterial due to sequence similarities (Lloyd et al., 2013). Some archaeal groups, such as Thaumarchaeota and Methanomicrobia, showed consistency between the two approaches (**Figure 9**). In contrast, Methanobacteria were observed mainly from predicted peptide data, and the “Miscellaneous Crenarchaeotal Group” (Kubo et al., 2012) was identified only in SSU rRNA data (**Figure 9**). To classify the predominant Archaea present in the sediment metagenomes, we analyzed the most abundant archaeal SSU rRNA sequences represented by long contigs (using selection criteria of ≥4X coverage and ≥1 Kb length; **Figure 10**). The highest number of contigs (6) meeting these criteria was observed in the BB metagenome, followed by YB-M (3) and YB-B (1).

### Thaumarchaeota

One relatively long contig from YB-B (YB-B-12 in **Figure 10**) was 97% identical (over 1477 bp) to the 16S rRNA gene from the freshwater thaumarchaeon *Nitrosoarchaeum limnia*. Read coverage for this contig was high (average of 65X coverage over the full length). Because *N. limnia* is a member of the ammonia-oxidizing archaea, we performed a HMMER3 search of the unassembled YB-B metagenome for reads corresponding to the functional gene for ammonia monooxygenase subunit A (*amoA*). Although only one full length (590 bp) *amoA* contig was annotated from the YB-B metagenome, it was 99% identical to several environmental *amoA* sequences recovered from rice paddies and soil, and 95.5 and 91.7% identitical to the *N. limnia* and *N. maritimus amoA* sequences, respectively. Furthermore, read coverage was 70X for this contig, which was very similar to that observed for the *N. limnia*-like 16S rRNA gene. Since both 16S rRNA and *amoA* genes are found in single-copy in the *N. limnia* genome, these data suggest that they were recovered from the same abundant representative of an *N. limnia*-like member of the Thaumarchaeota in YB freshwater sediments.

Thaumarchaeal *amoA* genes were also observed, in lower abundance, in YB-M and BB sediment metagenomes, with 4.1 and 4.6X coverage, respectively. These sediments are exposed to higher salinity water that does not penetrate into the freshwater regions further back in YB. This difference was reflected by the identity of sequences recovered from the three metagenomes. In YB-M, a 16S rRNA contig of 1165 bp (YB-M-206 in **Figure 10**) was 99 and 98% identical to 16S rRNA genes from *Candidatus* “Nitrosoarchaeum koreensis” and *Ca.* “Nitrosopumilus koreensis,” respectively. An *amoA* contig with a similar coverage (5X) was 93% identical to the *N. maritimus* gene. Similarly, the BB metagenome contained short 16S rRNA and *amoA* gene contigs with 93 and 95% identities to the corresponding genes from *N. maritimus*.

We used quantitative PCR (qPCR) to determine the abundance of the thaumarchaeal *amoA* gene in the sediment core samples used for metagenome analysis. The results indicated that the YB-B sample contained 6.49 × 10^7^ (SD 1.53 × 10^6^) *amoA* copies per gram of sediment, which was 29 and 18X higher than *amoA* copies in YB-M and BB samples, respectively. The qPCR results were in good agreement with the results from HMMER3 searches for SSU rRNA and *amoA* genes indicating that Thaumarchaeota populations were approximately 20X more abundant in the freshwater YB-B metagenome, compared to those from the more marine-influenced YB-M and BB samples.

### Novel and Ambiguous Archaeal Taxa

Thaumarchaeota represented 69 and 73% of the archaeal sequences identified in the YB-B metagenome as 16S rRNA genes and predicted peptides, respectively (**Figure 9**). In contrast, archaeal sequences identified in the BB and YB-M metagenomes were more diverse (**Figure 9**), with many corresponding to poorly characterized taxa without fully sequenced reference genomes. One group was classified by IMG/M-ER as Halobacteria (according to SSU rRNA gene and predicted peptide analyses, **Figure 9**). However, similarity-based searches indicated that the corresponding 16S rRNA genes shared the highest identity (98–99%) to PCR-amplified sequences from estuarine sediments (Abreu et al., 2001), and were only distantly related (82% identity) to well-characterized and fully sequenced Halobacteria from high-salt habitats. Another sequence group was classified as Thermoprotei; however, in contrast to well-characterized thermophilic representatives of this taxon, these sequences represented uncharacterized mesophilic archaea (“Thermoprotei-like Crenarchaeota” in **Figure 9**). One of the most poorly characterized groups, “Miscellaneous Crenarchaeota Group” (MCG), was also very abundant (representing ≥10% of archaeal 16S rRNA sequences) in YB-M and BB metagenomes (**Figure 9**). Five 16S rRNA gene contigs >1 Kb were identified from YB-M and BB metagenomes. Of these, two were classified as Thermoprotei-like, two as MCG, and one (discussed below) was related to sequences from the Methanomicrobia (**Figure 10**).

Another poorly characterized, but intriguing group identified by SSU rRNA gene sequences in BB and YB-M metagenomes was the so-called “7th order of methanogens” (Euryarchaeota), related to the Thermoplasmata and recently re-classified as “Methanoplasmatales” (Paul et al., 2012; Borrel et al., 2013). This group constituted >20% of all identified archaeal sequences in the BB metagenome (**Figure 9**), and is represented by two 16S rRNA contigs >1 Kb (with 34 and 14X coverage for BB-17 and BB-43, respectively, **Figure 10**) that placed phylogenetically with 16S rRNA gene sequences of characterized Methanoplasmatales. A long contig (with approximately 7X coverage) corresponding to *mcrA*, a specific biomarker for methanogenesis encoding methyl coenzyme M reductase A (Paul et al., 2012), was also found in the BB metagenome. This contig was 93% identical to the *mcrA* gene of a recently sequenced Methanoplasmatales representative, *Methanomassiliicoccus luminyensis*, isolated from human feces (Dridi et al., 2012). The difference in coverage between the 16S rRNA and *mcrA* contigs indicated that additional sequences of this novel group may have been present, but missed by our searches.

### Methanogenic and Methanotrophic Archaea

BB and YB-M metagenomes both contained an abundance of diverse sequences representing well-characterized methanogenic archaea, including Methanomicrobia and Methanobacteria, in addition to the Methanoplasmatales described above (**Figure 9**). The BB metagenome in particular, with approximately sixfold higher abundance of archaeal sequences, contained a variety of short (200–700 bp) 16S rRNA and *mcrA* contigs with coverage ranging from 2 to 9X and 86 to 99.5% identity to the corresponding genes from fully sequenced genomes of methanogenic archaea (i.e., Methanomicrobiaceae, Methanoregulaceae, Methanosaetaceae, and Methanosarcinaceae families).

One long (>1 Kb) 16S rRNA gene contig in the BB metagenome (BB-63 in **Figure 10**) related to Methanomicrobia was 93% identical to sequences from anaerobic methanotrophic archaea of the ANME-1 clade, which are capable of the anaerobic oxidation of methane (AOM; Meyerdierks et al., 2005). The sequence with the highest identity (97%) to the BB contig was recovered from the Yangtze River mudflats (Zeleke et al., 2013). A HMMER3 search also identified two *mcrA* contigs with closest identity (86–90%) to ANME-1 clones from a Mediterranean submarine mud volcano (Lazar et al., 2012). Since coverage was similar for both the 16S rRNA and the *mcrA* gene contigs (7.3 and 5.9X, respectively), it is possible that they represent the same ANME-1 group in the BB sediment.

### Methanogenesis-Related Functional Gene Categories

**FIGURE 11:**
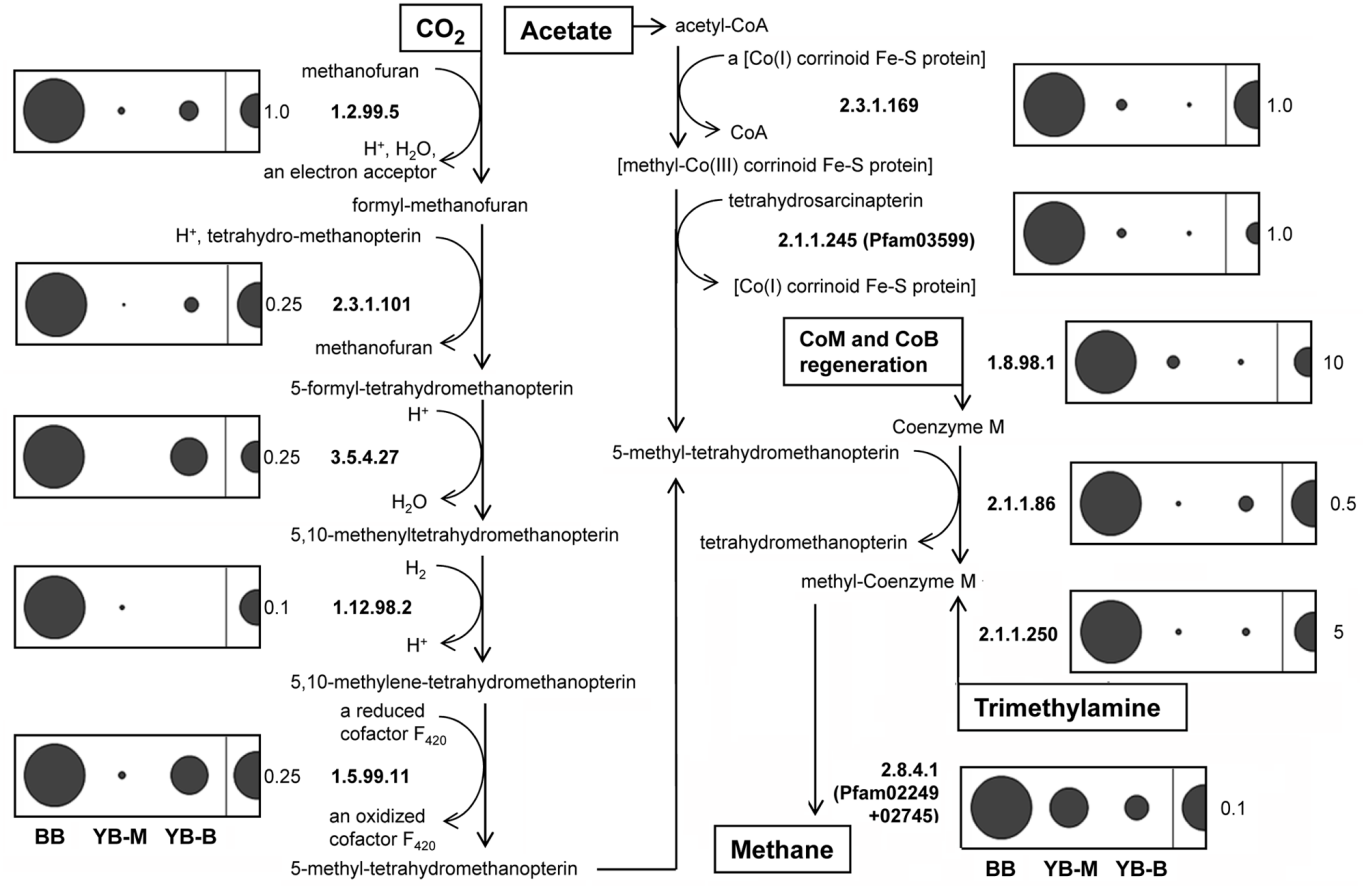
**Methanogenesis pathways (methanogenesis from CO_2_, acetate, and trimethylamine adapted from MetaCyc Pathways), and normalized abundance of the corresponding genes in sediment metagenomes.** The numbers represent enzymes based on the EC classification. The categories EC 1.12.98.2 and 1.5.99.11 have been recently transferred to EC 1.5.98.1 and 1.5.98.2, respectively. Abundance for a functional gene category was calculated as the number of hits to a given category normalized by the effective bacterial genome equivalents in the corresponding assembled metagenome. Abundance values are indicated by bubble width, with the scale shown as a half-bubble to the right in each panel (the number to the right shows the scale value, from 0.1 to 10 genes per the effective bacterial genome equivalent). Metagenome names are shown at the bottom left, they indicate the sampling locations: BB, Baker Bay; YB-M, Youngs Bay mouth; and YB-B, Youngs Bay back.

Many of the methanogenic archaea observed in the lateral bay samples corresponded to poorly characterized taxa. We therefore analyzed predicted peptide composition using functional gene classifications to identify metagenome metabolic properties. The functional categories involved in archaeal methanogenesis (**Figure 11**) were selected using the MetaCyc Pathway database (Caspi et al., 2012). In total, 11 key enzymes were highly enriched in the BB metagenome: on average 10 and 6.5X in comparison with YB-M and YB-B metagenomes, respectively (**Figure 11**). They consisted of the five key enzymes catalyzing methanogenesis from H_2_/CO_2_ (**Figure 11**, left panel), plus (i) two key enzymes of acetoclastic methanogenesis (methyltransferases Mt-cdh, C and D, EC: 2.3.1.169 and EC:2.1.1.245); (ii) the marker gene for methanogenesis from trimethylamine (trimethylamine methyltransferase Mb-mttB, EC:2.1.1.250); (iii) the gene catalyzing cofactor regeneration during methanogenesis (hdr, CoB-CoM heterodisulfide reductase, various subunits classified as EC:1.8.98.1) and (iv) two genes involved in the last common steps of methane production in the different pathways: coenzyme M methylation and reduction to methane (H4mpt, tetrahydromethanopterin *S*-methyltransferase EC:2.1.1.86, and mcrA, co-B sulfoethylthiotransferase; EC:2.8.4.1, pfam02249, 02745; **Figure 11**). Applying the *D*-rank approach (Markowitz et al., 2008), we determined that enrichment for 701 functional gene categories was significant in BB versus both YB-M and YB-B metagenomes (data not shown). Among these categories, the highest enrichment was observed for the methanogenesis-related *hdr* gene (29 and 36X, *p* < 0.009 for BB over YB-M and YB-B, respectively). In total, 6 out of 11 methanogenesis-related gene categories shown in **Figure 11** were significantly (>2.33, with *p*-values < 0.009) enriched in BB versus YB-M and YB-B.

### Methanotrophic and Syntrophic Bacteria

**FIGURE 12:**
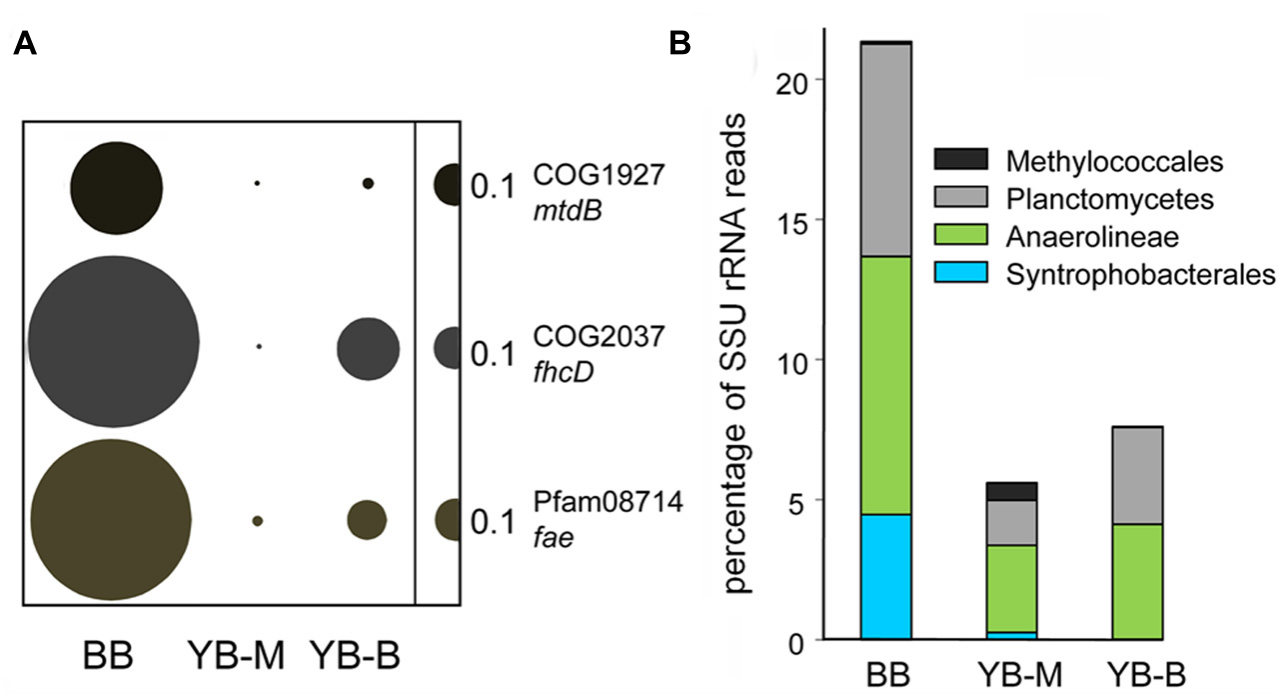
**(A)** Functional gene categories involved in methanotrophy/methylotrophy. The genes are mtdB, coenzyme F420-dependent *N*(5),*N*(10)-methenyltetra hydromethanopterin dehydrogenase; fhcD, formylmethanofuran:tetrahydromethanopterin formyltransferase; fae, formaldehyde-activating enzyme. Abundance of a functional gene category was calculated as the number of hits to a given category normalized by the effective bacterial genome equivalents in the corresponding assembled metagenome. Abundance values are indicated by bubble width, with the scale shown as a half-bubble to the right in each panel. **(B)** Relative phylogenetic abundance of putative methylotrophs and synthrophic bacteria are shown as the percentages of all SSU rRNA reads (selected by HMMER3 from unassembled metagenomes). Metagenome names are shown at the bottom, they indicate the sampling locations: BB, Baker Bay; YB-M, Youngs Bay mouth; and YB-B, Youngs Bay back.

Two of the identified marker genes [**Figure 11**; *hdr* (EC:1.8.98.1), and *mch* (EC:3.5.4.27)] are involved in both production and degradation of methane (Chistoserdova et al., 2004). This may explain the high numbers of the corresponding sequence hits observed, e.g., 2672 for *hdr* in the BB metagenome. In addition, several other markers of methylotrophy (Kalyuzhnaya et al., 2008) were enriched in the BB metagenome, with normalized abundances 10–33X higher than those in the YB-M metagenome (**Figure 12A**).

Enrichment of methylotrophic taxa was also detected in the YB-M metagenome from analysis of both SSU rRNA and peptides; most notably for the family Methylococcaceae (**Figure 12B**). However, a much greater enrichment was observed for putative Planctomycete methylotrophs (up to 8% of the total bacterial SSU rRNA reads) in the BB metagenome (**Figure 12B**). Certain members of the Planctomycetes are believed to carry out methano- and methylotrophy because they contain genes for C1 transfers mediated by methanopterin and methanofuran, similar to Proteobacteria methylotrophs (Chistoserdova et al., 2004). This result, together with the finding of abundant sequences from methane biosynthesis pathways, indicates that the methane produced in the sediments at the BB site was at least partially consumed there.

Enrichment was also seen in our data set for two major groups of syntrophic and semi-syntrophic organisms from the order Syntrophobacterales (Deltaproteobacteria; Narihiro and Sekiguchi, 2007) and the Anaerolineaceae family (Chloroflexi; Narihiro et al., 2012), respectively. Based on peptide data, the Syntrophobacterales order was enriched approximately 6.5X in the BB metagenome in comparison with YB-M metagenome. The Anaerolineaceae family represented 5.5% of all bacterial peptides in the BB metagenome, and was enriched 2.5X in comparison with the YB-M metagenome. Enrichment of both groups in the BB relative to the YB-M metagenome was even more apparent from the SSU rRNA data (17X, and 3X for Syntrophobacterales and Anaerolineaceae, respectively; **Figure 12**).

## Discussion

### Methanogenesis in Lateral Bay Sediments

The Columbia River and its major tributary, the Willamette River, have dissolved methane concentrations 1–2 orders of magnitude higher than open ocean waters (Anthony et al., 2012). Additional estuarine sources of methane have been identified at intermediate to high salinities, and they include intertidal mudflats (Middelburg et al., 2002). In this habitat, much of the organic matter degradation is carried out anaerobically by interacting groups of methanogenic archaea and SRB (O’Sullivan et al., 2013; Zeleke et al., 2013). Complementary analysis of our sediment samples by Illumina paired-end sequencing and *de novo* genome assembly of 56 *Methanosarcina mazei* isolates revealed phenotypically distinct clades of *M. mazei* with different methane production rates during growth on trimethylamine (Youngblut et al., 2015). This observation is consistent with the maintenance of diversity in lateral bay sediments through ecological differentiation. Our comparison of lateral bay metagenomes likewise found that SSU rRNA genes of methanogens (**Figure 9**), and predicted peptides from functional gene categories required for producing methane from CO_2_, acetate and trimethylamine, were highly enriched in the BB metagenome (**Figure 11**). In fact, methanogenesis was the most obvious microbial metabolism distinguishing BB from YB-M and YB-B metagenomes (3–30X enrichment of the corresponding functional gene categories observed in BB). Methanogenic archaea were also found to dominate the near-surface brackish estuarine sediments of the Yangtze River in China (Zeleke et al., 2013). Interestingly, there were also abundant (but poorly characterized) putative methylo/methanotrophs from the Planctomycetes phylum in the BB metagenome. Our data suggest that both methanogenesis and methanotrophy occur in BB sediments. Many of the genes encoding components of the methanogenic pathway have been found in genomes of bacteria and archaea involved in aerobic and anaerobic methanotrophy (Chistoserdova et al., 1998, 2004). Methane produced in this environment may be oxidized by anaerobic methanotrophs (ANME) using sulfate as electron acceptor (Knittel and Boetius, 2009), or diffuse across the oxic/anoxic interface to be oxidized aerobically. Alternatively, methane produced in the lateral bay sediments may be advected into the main channel during the ebb stages of the tidal cycle. Previous studies have shown that gasses are transported from intertidal marshes into estuaries with the ebb tide, and may surpass the eﬄux of dissolved organic matter (Cai et al., 1999). While net methane emission into the atmosphere from the Columbia River estuary seems likely (Middelburg et al., 2002), methane measurements indicate that biological consumption does occur (Sansone et al., 1999).

### Methanogenesis and Syntrophic Bacteria

Sulfate-reducing bacteria are a diverse group of obligate, anaerobic bacteria that use sulfate as the terminal electron acceptor during the oxidation of a variety of electron donors, many of them organic (for review see Plugge et al., 2010). SRB are known to outcompete methanogenic archaea for substrates such as H_2_ and acetate; thus, high sulfate concentrations in ocean tidal water influx favor sulfate reduction as the major process for anaerobic organic matter utilization (Oremland and Polcin, 1982; Leloup et al., 2009). In our work, SRB and the obligate hydrogenotrophic methanogens (i.e., Methanomicrobia) were found in the same metagenome. One possible explanation is the fine-scale vertical separation of these two groups in sediments was overlooked when the upper 6 cm were collected as a single sample.

Other methanogens may co-exist with SRB because the latter do not compete for methanogenic substrates such as methanol, trimethylamine, or methionine (Oremland and Polcin, 1982; Leloup et al., 2009). Moreover, some SRB, i.e., *Desulfovibrio vulgaris*, are capable of metabolic shifting between two lifestyles: from sulfidogenic growth to syntrophic growth in association with the methanogen *Methanosarcina barkeri* (Plugge et al., 2010). In the presence of sulfate, SRB carry out sulfidogenesis and oxidize the products of primary fermentations to CO_2_. Once sulfate is depleted, they ferment organic acids and alcohols, producing hydrogen, acetate and carbon dioxide, and subsequently rely on hydrogen- and acetate-scavenging methanogens to convert these compounds to methane (Plugge et al., 2010). This syntrophy is widespread in marine and estuarine environments, since it allows SRB to survive upon depletion of sulfate (which occurs every tidal cycle; Leloup et al., 2009; Plugge et al., 2010). SRB have been found to be abundant in zones with high methane production (Leloup et al., 2009).

Both YB-M and BB sediments are exposed to elevated sulfate concentrations periodically with the tides. Consistent with this, abundant sequences representing SRB communities were present at both locations (with the relative proportion being higher in BB than in YB-M, **Figure 9**). However, only in BB sediments did the SRB appear to co-exist with an abundant and diverse community of methanogenic archaea. Even though the Youngs Bay (YB-M) sediments have 3X more organic matter than BB (**Table 2**), it may be that the sources and specific types of organic matter present are more important. For example, it was noted (Macdonald and Winfield, 1984) that clumps of the marine brown alga *Fucus distichus edentatus*, an inhabitant of intertidal zones on rocky shores, commonly co-occurred within stands of sedges (*Carex lyngbyei*) in BB only. These and other seaweeds are noted for the relatively high amounts of trimethylamines associated with both live and decaying cells (Kneifel, 1979). Recent evidence points to obligate, H_2_-dependent methylotrophic methanogenesis and consumption of a diversity of methylated compounds by members of the Thermoplasmatales (Borrel et al., 2013), a particularly abundant group in the BB metagenome. Thus, the presence of these algae may play a role in the selective enrichment for methylotrophic methanogens in BB sediments. The BB metagenome was also enriched for syntrophic and semi-syntrophic bacteria from the order Syntrophobacterales (Narihiro and Sekiguchi, 2007) and the family Anaerolineaceae (Narihiro et al., 2012), which are known to be involved in anaerobic degradation of fatty acids and other carbohydrates and generation of substrates for methanogenesis (reviewed in Narihiro et al., 2012).

### Evidence for the Erosion of Lateral Bay Sediments and Trapping by ETM

Many of the same taxonomic groups found in the lateral bay sediment metagenomes in this study were previously found enriched in the large particulate fraction of ETM water samples, relative to the free-living, smaller-size fraction (Smith et al., 2013). These strict and facultative anaerobes included archaeal methanogens together with syntrophic bacterial taxa (i.e., SRB and the Anaerolineaceae family); and the Mn/Fe-reducing genera, *Pelobacter* and *Geobacter*. Unfortunately, the similarity-based peptide annotations used for taxonomic analyses could not unequivocally determine whether the ETM and lateral bay sediments were populated by the same organisms (at the species level), or whether sequences recovered from these two habitats represented related, but distinct members of the same family and/or genus. In addition, because our analysis was not carried out on full-length sequences, but rather on shorter fragments that were below a 97% identity threshold and sequence complexity was very high, it was not possible to determine whether SSU rRNA operational taxonomic units (OTUs) present in the water and in the sediments represented the same taxa. Nevertheless, the presence of taxonomically overlapping microbiota in ETM particles and lateral bay sediments may indicate particle transport and ‘seeding’ of the water with facultative and strict anaerobes from the sediments. This particle exchange would be driven by tidal forces, and possibly enhanced during spring tides and at times of low river discharge, such as during late summer/fall. Altogether, these data suggest potential erosion of lateral bay sediments, followed by their retention in the estuary in ETM, effectively enriching anaerobic microorganisms in the oxygenated water through the existence of particle-associated anoxic microzones (Smith et al., 2010, 2013).

### Particle Deposition from the Mainstem Estuary into Lateral Bays

Our simulation analysis indicated that the water masses originating from the continental shelf and plume regularly entered Youngs Bay in relatively high volumes. Thus, particulate matter, when present, may have settled onto the sediments near the mouth of Youngs Bay during slack tides. Analysis of microbial community composition in fact suggested large-scale deposition of several diatom taxa, including the pelagic marine *Odontella*, and brackish water *P. laevis*. Previous observations suggested a shift of phytoplankton assemblages toward marine species under late summer conditions of low river flow, particularly near the mouth of the estuary (Breckenridge et al., 2014). Consistent with our sequence data, several prolonged periods of coastal ocean upwelling with associated phytoplankton bloom development were observed for ∼1.5 months prior to our sample collections (**Figure 2**). Continuous SATURN station observations showed that high-salinity and high-nitrate water masses from the upwelling-influenced coastal ocean were periodically observed in the main South Channel of the Columbia River estuary in July–August 2011, transporting elevated concentrations of chl *a* (**Figure 3A**). At the same time, blooms of *Mesodinium* sp. were not detected during August 2011. Freshwater chl *a* concentrations were also consistently low (**Figure 3A**, bottom panel), indicating that the phytoplankton detected in our YB-M sediment sample originated in marine waters. Together with our sequence data, these results suggest that marine diatoms were transported into the estuary with the tides, and deposited as perished and decaying cells in the bottom sediments near the YB mouth.

Previous microscopic surveys of shallow lateral bay sediments (Frey et al., 1984; Simenstad et al., 1984) revealed assemblages of mostly benthic diatoms, and did not find marine planktonic taxa in the upper layers of sediment, even in the samples from locations adjacent to the Columbia River mouth. In contrast, multiple marine diatoms (i.e., *Asterionellopsis glacialis*) have been identified by microcopy in the estuarine water column at the mouth of Columbia River (Breckenridge et al., 2014). Possibly, prior to sediment deposition, live diatom cells were lysed at the estuarine freshwater-seawater interface (Sherwood et al., 1984), with rapid deterioration of the thin silica frustules used to distinguish these species under the microscope (Simenstad et al., 1984). Our data provide indirect evidence of diatom degradation, since diatom chloroplast rRNA genes, and sequences corresponding to the chloroplast-encoded photosystem I psaA/psaB and RuBisCo large subunit proteins were abundant, whereas the nuclear-encoded RuBisCo small subunit sequences were not found. Protection of multiple chloroplast membranes is proposed to make chloroplast DNA more stable in the environment than nuclear DNA upon cell death (Bedoshvili et al., 2009; Smith et al., 2013). Thus, detrital deposition of phytoplankton observable at the chloroplast DNA level could pass undetected by microscopic analysis.

Interestingly, the deposition of decaying bloom particles had a dramatic effect on the sediment bacterial community composition, apparently leading to significant enrichment of Bacteroidetes (>50% of all identified bacterial peptides) in the YB-M sample (**Figures 6** and **8A**). Bacteroidetes/Flavobacteria are known to be abundant during periods of high primary production (Williams et al., 2012), and members of these taxa degrade phytoplankton polysaccharides (Kirchman, 2002). For example, in the North Sea their abundance was increased fivefold upon commencement of algal blooms (Teeling et al., 2012). In our previous work we also observed enrichment of Flavobacteria in diatom-replete euphotic zone water samples, compared to samples from the deep ocean containing few diatom sequences (Smith et al., 2013). Bacteroidetes genes coding for carbohydrate-active enzymes (CAZymes) have been linked to particle utilization (Teeling et al., 2012). Our analysis of CAZyme functional categories in the sediment metagenomes showed a dramatic enrichment (>70%) of abundant CAZyme genes in the YB-M metagenome (**Figure 7B**). It may be that the Bacteroidetes/Flavobacteria in the YB-M sample were indigenous to the sediments, with their growth and activity selected for by deposition events. Alternatively, they may have colonized the diatom biomass in the coastal ocean or the estuary during transport, and were deposited onto the bottom sediments along with detrital particles. Thus, during periods of high primary production (such as late summer) lateral bay sediments of the lower Columbia River estuary may serve as repositories of biogenic particles, and the allochthonous particle influxes may provide a major force shaping microbial community composition and activities.

## Conclusion

Our metagenomic data revealed BB as a potentially important site for methanogenesis in the Columbia River estuary. This putative methylotrophic methanogen community may play a major role in distinguishing the microbial biogeochemistry of BB from similarly salinity-influenced regions of Youngs Bay.

Our data additionally provide support for reciprocal particle exchange between the lateral bays and the mainstem water column. These exchange events can dramatically alter the microbial community composition and activities, for example enriching specifically for diatom-degrading bacteria in localized “hotspots” within lateral bay sediments. Several lines of evidence also suggested that lateral bay sediments may be involved in particle “seeding” of ETM, resulting in enrichment of anaerobic microorganisms in the oxygenated water column (Crump et al., 1999; Smith et al., 2010, 2013; Bräuer et al., 2011; Simon et al., 2014). Analysis of the lateral bay sediment metagenomes supports these conclusions, revealing related taxonomic groups. Currently we are expanding the pilot study through metagenome analysis of eight additional sediment samples from Baker, Youngs and Cathlamet Bays of the lower estuary. This should increase our ability to interpret complex abundance patterns observed for bacterial taxa, to relate specific microbial metabolisms to biogeochemical measurements, and ultimately, to better define the roles of lateral bays in the generation of net ecosystem metabolism in the lower Columbia River estuary.

## Author Contributions

MS, LH, and HS conceived and designed the study, collected the environmental samples, performed the experiments, conducted data analysis, and wrote the manuscript. RD and NY performed metagenome assembly, annotation, and comparative data analyses. TK and AB performed numerical simulations of water mass transport. RW, WM, and BT contributed to the data analysis, manuscript writing and editing.

## Conflict of Interest Statement

The authors declare that the research was conducted in the absence of any commercial or financial relationships that could be construed as a potential conflict of interest.

## Footnotes

1 http://www.stccmop.org/datamart/observation_network

2 http://www.arb-silva.de/

3 http://hmmer.janelia.org/

4 http://lyorn.idyll.org/∼t/khmer/index.html

5 https://img.jgi.doe.gov/cgi-bin/mer/main.cgi

6 http://las.pfeg.noaa.gov/oceanWatch/oceanwatch.php
